# Eicosapentaenoic acid ethyl ester, cardiac metabolomic and lipidomic signatures, and cardioprotection in myocardial infarction

**DOI:** 10.1093/eurheartj/ehag187

**Published:** 2026-04-17

**Authors:** Sebastià Alcover, Lisaidy Ramos-Regalado, Natàlia Muñoz-García, Gabriela O Girón, Sergi Otero, Sergi López, Teresa Padro, Lina Badimon, Gemma Vilahur

**Affiliations:** Institut de Recerca Sant Pau (IR SANT PAU), Sant Quintí 77-79, 08041 Barcelona, Spain; Facultat de Biologia, Universitat de Barcelona, 08028 Barcelona, Spain; Institut de Recerca Sant Pau (IR SANT PAU), Sant Quintí 77-79, 08041 Barcelona, Spain; Facultat de Biologia, Universitat de Barcelona, 08028 Barcelona, Spain; Institut de Recerca Sant Pau (IR SANT PAU), Sant Quintí 77-79, 08041 Barcelona, Spain; Facultat de Biologia, Universitat de Barcelona, 08028 Barcelona, Spain; Institut de Recerca Sant Pau (IR SANT PAU), Sant Quintí 77-79, 08041 Barcelona, Spain; Facultat de Biociències, Universitat Autònoma de Barcelona, 08193 Barcelona, Spain; Institut de Recerca Sant Pau (IR SANT PAU), Sant Quintí 77-79, 08041 Barcelona, Spain; Facultat de Biociències, Universitat Autònoma de Barcelona, 08193 Barcelona, Spain; Institut de Recerca Sant Pau (IR SANT PAU), Sant Quintí 77-79, 08041 Barcelona, Spain; Institut de Recerca Sant Pau (IR SANT PAU), Sant Quintí 77-79, 08041 Barcelona, Spain; Centro de Investigación Biomédica en Red Enfermedades Cardiovasculares Instituto de Salud Carlos III, 28029 Madrid, Spain; Institut de Recerca Sant Pau (IR SANT PAU), Sant Quintí 77-79, 08041 Barcelona, Spain; Centro de Investigación Biomédica en Red Enfermedades Cardiovasculares Instituto de Salud Carlos III, 28029 Madrid, Spain; Facultat de Medicina, Universitat de VIC-UCC and Cardiovascular Research Foundation for Health Prevention and Innovation (FICSI), 08017 Barcelona, Spain; Institut de Recerca Sant Pau (IR SANT PAU), Sant Quintí 77-79, 08041 Barcelona, Spain; Centro de Investigación Biomédica en Red Enfermedades Cardiovasculares Instituto de Salud Carlos III, 28029 Madrid, Spain

**Keywords:** Animal model, Myocardial infarction, Infarct size, Eicosapentaenoic acid ethyl ester, Cardioprotection, Inflammation, Metabolomics, Lipidomics

## Abstract

**Background and Aims:**

The cardiovascular benefits of eicosapentaenoic acid ethyl ester (EPA-E) in MACE reduction might extend beyond its triglyceride (TG)-lowering effects. However, the mechanism behind remains unknown. This study investigated whether EPA-E exerts cardioprotection in the setting of myocardial infarction (MI).

**Methods:**

Hypertriglyceridaemia (HTG) was induced in male rats by a high-sucrose diet (>175 mg·dL^−1^; baseline). Hypertriglyceridaemia rats were administered for 2 weeks high-dose EPA-E supplementation (HTG + EPA-E; *n* = 21) or placebo (HTG; *n* = 27) in combination with the diet. A control diet group (CD; *n* = 15) was included. After 2 weeks, MI was induced by transient coronary ligation and animals were sacrificed 24 h thereafter for infarct size, molecular, histological, and -omic analyses. Echocardiography was performed.

**Results:**

HTG + EPA-E animals displayed lower circulating TG levels and better cardiac function as compared to HTG group. HTG + EPA-E animals showed smaller infarcts (*P* < 0.05 vs both groups), and MI-related mortality was 29% in EPA-E-treated animals (reaching levels comparable to those of the CD group), whereas it was 56% in HTG animals (*P* = 0.07). No correlation was observed between circulating TG levels and MI-related infarct size or mortality. Cardiac function was similarly deteriorated in all animals post-MI. Eicosapentaenoic acid ethyl ester treatment was associated with lower TG-related cardiolipotoxicity, apoptosis, fibrosis, oxidative stress, pro-inflammatory cell recruitment and less mitochondrial dysfunction in the infarcted heart. EPA-E treatment favoured a pro-resolving response and improved metabolomic and lipidomic profiles both systemically and in the infarcted heart.

**Conclusions:**

Eicosapentaenoic acid ethyl ester treatment exerts direct cardioprotective effects and ameliorates cardiac metabolic and lipidomic signatures after MI regardless of its circulating TG-lowering effects.

## Structured Graphical Abstract

**Structured Graphical Abstract:**
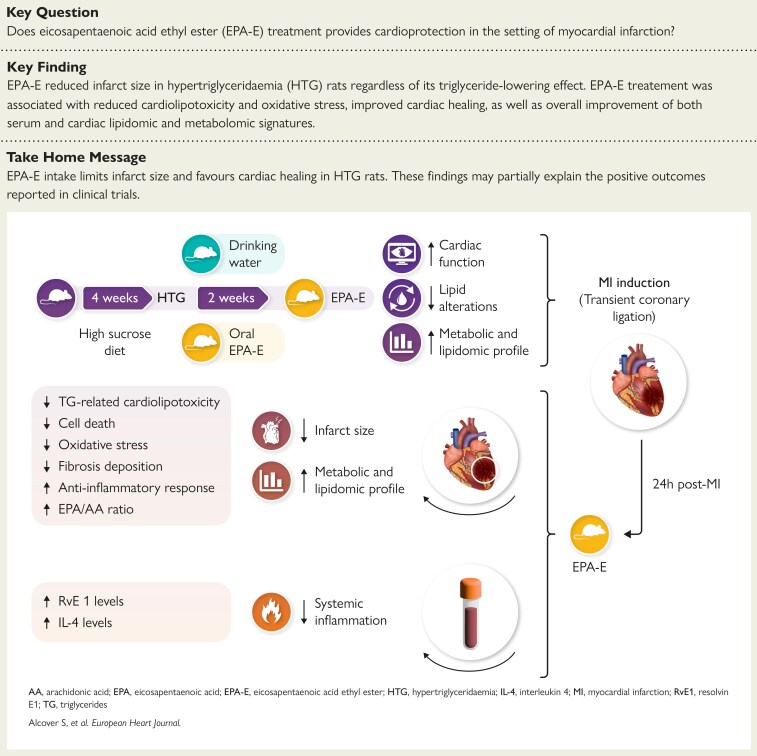

**See the editorial comment for this article ‘Eicosapentaenoic acid ethyl ester cardioprotection: beyond triglycerides’, by Tarec K. Elajami *et al.*, https://doi.org/10.1093/eurheartj/ehag287.**

Translational perspective This study highlights the potential of eicosapentaenoic acid ethyl ester (EPA-E) as a cardioprotective therapy extending beyond its lipid-modulating benefits. As such, EPA-E may limit infarct severity regardless of its hypotriglyceridaemic effects.

## Introduction

Hypertriglyceridaemia (HTG) is highly prevalent (29.6%) across Europe.^1^ Epidemiological studies have identified elevated triglycerides (TG) levels as an independent risk factor for cardiovascular (CV) disease (CVD), with a 5.1-fold increased risk of myocardial infarction (MI) and a 2.2-fold increase in all-cause mortality.^2^ Furthermore, epidemiologic studies have shown that elevated TG levels correlate with higher CV residual risk in patients with optimal and controlled LDL cholesterol (LDL-C) levels.^3–5^ Moreover, genetic,^6,7^ Mendelian randomization,^8^ and clinical studies^9,10^ support that high TG levels play a causal role in the development of atherosclerotic cardiovascular disease (ASCVD).

Given the key role of TG on CV residual risk, there has been an increasing interest in identifying effective TG-lowering therapeutic approaches. Although lower TG levels are associated with reduced ASCVD event risk, most CV outcomes trials of TG-lowering therapies (such as those involving niacin^11,12^ and fibrates^13,14^) have failed to demonstrate significant reductions in CV endpoints, despite improvements in patients’ lipid profiles.^11,15^ REDUCE-IT^16^ was the first clinical trial to demonstrate a reduction in major adverse cardiovascular events in eicosapentaenoic acid ethyl ester (EPA-E; a pure ω-3 fatty acid)–treated patients with HTG and statin therapy on board.^17^ In light of these findings, the 2019 ESC guidelines recommend considering EPA-E treatment (Class IIa, Level of Evidence B) for high-risk HTG patients, particularly in combination with statins.^1,18^ However, the underlying mechanisms driving these beneficial outcomes remain incompletely understood, as they cannot be solely attributed to their TG-lowering effect^19^ and are evident regardless of baseline circulating TG levels.^16^ The EVAPORATE trial^20^ further demonstrated that EPA-E reduces features of atheromatous plaque vulnerability and enhances coronary flow.

Multiple experimental studies and clinical trials have supported the ability of EPA-E to modulate the TG-rich lipoprotein (TRL) metabolism in patients with metabolic disorders,^21,22^ preserve cardiac function in patients with chronic heart failure and dyslipidaemia,^23^ and improve outcomes in hypertensive rat models.^24^ Additionally, EPA-E has been shown to modulate inflammatory responses in CD4^+^ T cells *in vitro*,^25^ to attenuate inflammation,^26^ and to reduce collagen deposition^26,27^ in a rodent model of MI. However, whether EPA-E treatment exerts direct cardioprotection in a background of HTG remains to be addressed. In the present study, we demonstrate, in a MI rat model of HTG, that EPA-E administration limits infarct size (IS) and enhances cardiac healing post-MI. Furthermore, we explore the underlying mechanisms behind these benefits by combining molecular, histological, metabolomic, and lipidomic analyses.

## Methods

The detailed description of the methodology for this study is provided in the Supplementary data.

The study design was reviewed and approved by the Institutional Animal Care and Use Committees (procedure #152) and authorized by the Animal Experimental Committee of the local government by the Spanish law (RD 53/2013) and European Directive 2010/63/EU. This study complies with NIH animal care guidelines, adheres to ARRIVE 2.0 standards, and follows current recommendations for rigour in preclinical cardioprotection research, while upholding the 3Rs principle and minimizing animal use.

### Experimental design

**Figure 1:**
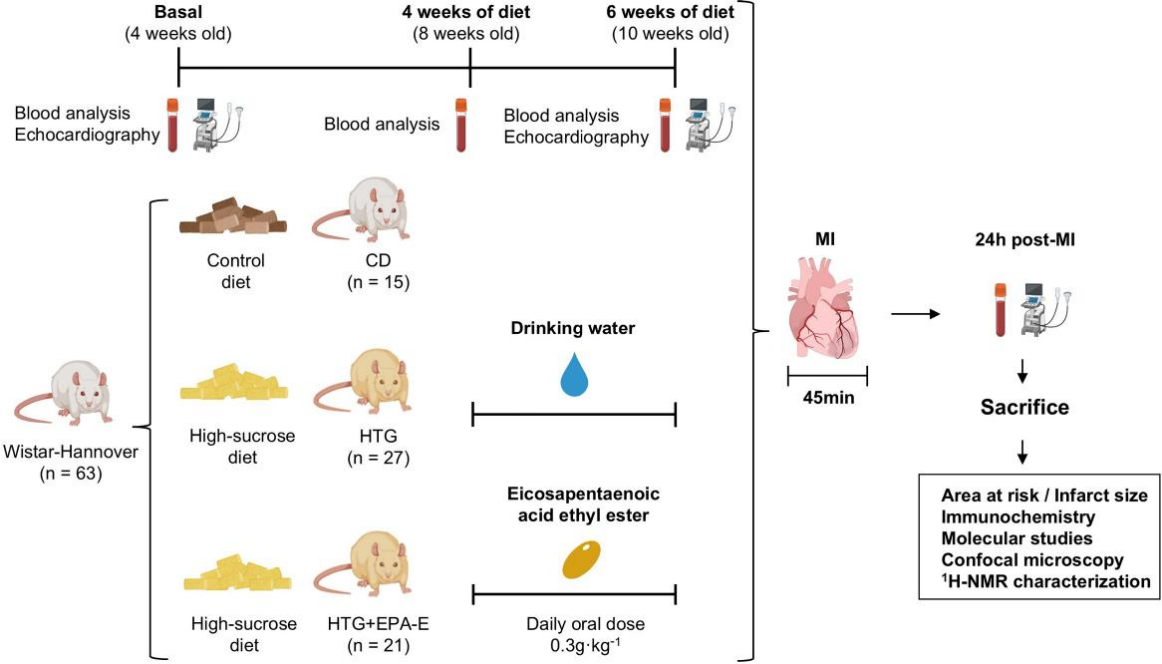
Experimental design. Male Wistar-Hannover rats were randomly allocated before baseline measurements into three groups: (i) control diet group (CD; *n* = 15); (ii) a high-sucrose diet group (HTG; *n* = 27); and (iii) a high-sucrose diet EPA-E-treated group (HTG + EPA-E; *n* = 21). Serum samples were taken at baseline, Week 4, Week 6, and 24 h post-MI to monitor HTG development. At 4 weeks, after the assessment of HTG condition, HTG + EPA-E group received a daily oral dose of EPA-E at 0.3 g·kg⁻¹ for 2 weeks and HTG group received an equivalent volume of drinking water. At 6 weeks, MI was induced by transient ligation of the LAD coronary artery for 45 min and then animals were allowed to recover. All animals were sacrificed 24 h post-MI for infarct size assessment and tissue and blood sample collection. CD, control diet group; HTG, hypertriglyceridaemia group; EPA-E, eicosapentaenoic acid ethyl ester-treated group; MI, myocardial infarction; LAD, left anterior descending

Male Wistar-Hannover rats (*n* = 63; 125–150 g; 4 weeks old) were randomly allocated before baseline measurements into three groups: (i) control diet group (CD; *n* = 15; 28.4% alfalfa, 6.4% sugars); (ii) a high-sucrose diet group (HTG; *n* = 27; 2.8% alfalfa, 56.2% sugars); and (iii) a high-sucrose diet EPA-E-treated group (HTG + EPA-E; *n* = 21; 2.8% alfalfa, 56.2% sugars). All animals were maintained on their respective diets for 6 weeks with *ad libitum* access to food and water (*Figure 1*). Diet compositions are detailed in Supplementary data online, *Table S1*. Hypertriglyceridaemia was confirmed at 4 weeks based on fasting TG levels of >175 mg·dL^−1^, according to the most recent European guidelines^18^ and the TG threshold used to stratify patients in clinical trials.^28^ In addition, the high-sucrose-fed animals exhibited a 2.8-fold increase in TG relative to the CD group, a magnitude of elevation that closely mirrors that described in the hereditary HTG rat model.^29^

After HTG confirmation, the HTG + EPA-E group received EPA-E (0.3 g·kg⁻¹/day, 99% purity) for 2 weeks, whereas the HTG group received an equivalent volume of drinking water. The EPA-E dose corresponded to the human 4 g/day prescription, adjusted for BSA.^16^ Thereafter, MI was induced by a 45 min occlusion of the left anterior descending artery (LAD), and animals were euthanized 24 h later for IS and sample collection. Blinded alphanumeric coding was used for all molecular, histological, and analytical procedures. Echocardiography was performed at baseline, Week 6, and 24 h post-MI. Analyses included only animals that survived the entire experimental procedure. The details of the samples evaluated across the different analyses are provided in Supplementary data online, *Table S2*.

### Transthoracic echocardiography

All animals underwent transthoracic echocardiography, as performed in our previous study.^30^ We assessed left ventricle (LV) ejection fraction (LVEF), LV shortening fraction (LVSF), LV anterior wall thickness in systole (LVAWs), LV anterior wall thickness in diastole (LVAWd), LV internal diameter in systole (LVIDs), LV internal diameter in diastole (LVIDd), LV mass, LV end-systolic volume (LVESV), and LV end-diastolic volume (LVEDV) (*n* = 12–15 animals/group; Supplementary data online, *Table S2*). Diastolic dysfunction was determined by isovolumic contractile time (IVCT), isovolumic relaxation time (IVRT), mitral valve ejection time (MVET), and *E* wave/*A* wave (*E/A* ratio). Finally, LV myocardial performance index (LVMPI) was calculated to evaluate overall cardiac function. Three representative cardiac cycles were evaluated for each determination, and heart rate (HR) was continuously monitored^31^ (*n* = 9–15 animals/group; Supplementary data online, *Table S2*).

### Myocardial infarction model and infarct size assessment

Surgeries were staggered weekly (six rats/week), with alternating group order to avoid procedural bias and maintain a uniform 14-day EPA-E treatment period. Myocardial infarction induction and IS assessment were performed as previously described.^30^ At Week 6, rats underwent thoracotomy and 45 min of transient LAD coronary artery ligation to induce MI. After 24 h, LAD was re-occluded and reperfused with Evans Blue to identify the area at risk (AAR). Hearts were excised, sectioned, and slices (four 2-mm-thick sections) were collected for molecular, IS, and histological analyses (*n* = 11–14 animals/group; Supplementary data online, *Table S2*).

Survival rates were assessed across two distinct phases: (i) the ischaemic phase, defined as the 45 min LAD occlusion period, and (ii) the acute post-MI phase, measured from LAD reperfusion to the time of sacrifice (24 h post-MI) (*n* = 15–27 animals/group; Supplementary data online, *Table S2*).

### Cardiac molecular fingerprint

Ischaemic myocardium (delineated by Evans Blue negative staining) was collected and frozen for mRNA and protein extraction.

#### Transcript levels

We determined cardiac gene expression levels of apoptosis (*Becn1*, *Casp3*), fibrosis (*Col1a1*, *Col3a1*), and inflammation (*Tlr4*, *Rptor*) related markers assessed by RT-PCR. qPCR was performed in duplicate and gene expression was normalized to HPRT1 (*n* = 10–15 animals/group; Supplementary data online, *Table S2*).

#### Protein expression and activation

We determined markers of apoptosis (cleaved and total caspase-3; C-CASP3/Total-CASP-3), antioxidant capacity (glutathione peroxidase 1; GPX1), autophagy/lipid peroxidation [protein kinase B (PKB); p-AKT, AKT], and mitochondrial homeostasis (optic atrophy protein 1; OPA1, dynamin-related protein 1; DRP1). Mitochondrial proteins were isolated following established protocols.^32^ Band intensities were normalized using a common reference and total protein Ponceau staining. Results were expressed in arbitrary units (a.u.) (*n* = 10–15 animals/group; Supplementary data online, *Table S2*).

### Immunohistochemical analyses

Serial 5 μm paraffin-embedded sections from infarcted myocardial tissue were processed for Masson’s trichrome staining to assess collagen volume fraction (CVF; from five random fields per animal), 4-hydroxynonenal (4-HNE) staining for lipid peroxidation, and immunohistochemistry for neutrophils (anti-elastase), macrophages (CD68), and CD206^+^ macrophages. Images were acquired using SlideViewer and analysed with ImageJ; 4-HNE and cell counts were measured in ten random fields per animal (*n* = 9–15 animals/group; Supplementary data online, *Table S2*).

### Analysis of apoptosis execution by TUNEL fluorescence assay

Apoptosis in the infarcted myocardial tissue was assessed using the TUNEL assay and imaged with a Leica inverted confocal microscope. Quantification was performed in four randomly selected fields per heart (*n* = 11–14 animals/group; Supplementary data online, *Table S2*).

### ^1^H-NMR characterization of the infarcted myocardial tissue and serum

Nuclear magnetic resonance (NMR) spectroscopy was conducted on infarcted myocardial tissue and serum samples to identify EPA-E-related metabolic and lipid alterations. Aqueous and lipid extracts were prepared via a modified Folch extraction; aqueous fractions were lyophilized and reconstituted in deuterated phosphate buffer with TSP, whereas lipid extracts were dissolved in deuterated chloroform. Spectra acquired on a 600 MHz Bruker Avance Neo system were processed using Dolphin and LipSpin to quantify metabolites and lipid species (*n* = 8–11 animals/group; Supplementary data online, *Table S2*). Remnant cholesterol (RM-C) serum levels were calculated as VLDL-C + IDL-C (mg/dL). The complete list of metabolites and lipids analysed is presented in Supplementary data online, *Table S3*.

### Targeted UHPLC-MS methodology for infarcted myocardial tissue

The targeted UHPLC-MS workflow involved N₂-drying of lipid extracts, reconstitution in isopropanol, and chromatographic separation on a C18 column prior to negative ESI-SIM detection of AA (arachidonic acid) and EPA (eicosapentaenoic acid) with a Vanquish Horizon-Orbitrap ID-X system. Data were processed using Xcalibur Quan Browser (*n* = 10–11 animals/group; Supplementary data online, *Table S2*).

### Systemic resolvin and cytokine levels

Serum concentrations of resolvin E1 (RvE1) and IL-4 were determined using specific ELISA kits (*n* = 8–9 animals/group; Supplementary data online, *Table S2*).

### Biochemical and haematological follow-up

Blood was collected at baseline, Weeks 4 and 6, and 24 h post-MI for serum isolation. Triglyceride, total cholesterol (TC), and glucose were measured using standard enzymatic assays (*n* = 12–15 animals/group; Supplementary data online, *Table S2*). Serum non-esterified fatty acids (NEFA) were quantified using a specific enzymatic colourimetric assay (*n* = 9–11 animals/group; Supplementary data online, *Table S2*).

### Statistical analysis

All analyses were performed blindly. Data are presented as mean ± SD. Survival was evaluated with Kaplan–Meier curves and log-rank (Mantel–Cox) testing, and Cox regression [SPSS Statistics (version 27; IBM Statistics)] was used to estimate hazard ratios (HzR) and 95% confidence intervals (CI). Linear regression analyses were performed to investigate the association between different parameters. Normality was assessed by Shapiro–Wilk. When normality could be assumed, comparisons were analysed by two-tailed *t*-test, one-way ANOVA, or two-way ANOVA for repeated measures, followed by Tukey’s *post hoc* test. When normality was not met, the Mann–Whitney or Kruskal–Wallis tests with Dunn’s *post hoc* correction were applied. Homogeneity of variances was tested using Brown–Forsythe. When variances were unequal and sample sizes differed, Welch ANOVA with Games-Howell *post hoc* test was applied. Mixed-effects models were employed when missing values or variance heterogeneity precluded two-way ANOVA approaches. All *P*-values reported in the figures and text correspond to adjusted *P*-values obtained after these corrections. Outliers were identified using the ROUT method (*Q* = 1%). *P* < 0.05 was considered to indicate statistically significant differences using GraphPad software.

In partial least squares discriminant analysis (PLS-DA), metabolites and lipids with >50% of missing values were removed from analysis. Values below the limit of detection (LoD) were imputed using the default LoD procedure, by assigning a value corresponding to 1/5 of the minimum positive feature value. Data were then Pareto-scaled before PLS-DA. Model robustness was assessed using a 1000-fold permutation test of the between/within (B/W) separation distance to minimize the possibility that the observed PLS-DA separation occurred by chance. Moreover, five-fold cross-validation approach, selected based on sample size constraints, was employed to evaluate the model’s goodness of fit (*R*²) and predictive ability (*Q*²), which correspond to the average metrics automatically calculated across the five validation folds by the platform. Variable importance was determined using VIP and coefficient scores. PLS-DA analysis and validation were done using MetaboAnalyst.

## Results

### Impact of eicosapentaenoic acid ethyl ester on the atherogenic lipid profile

**Figure 2:**
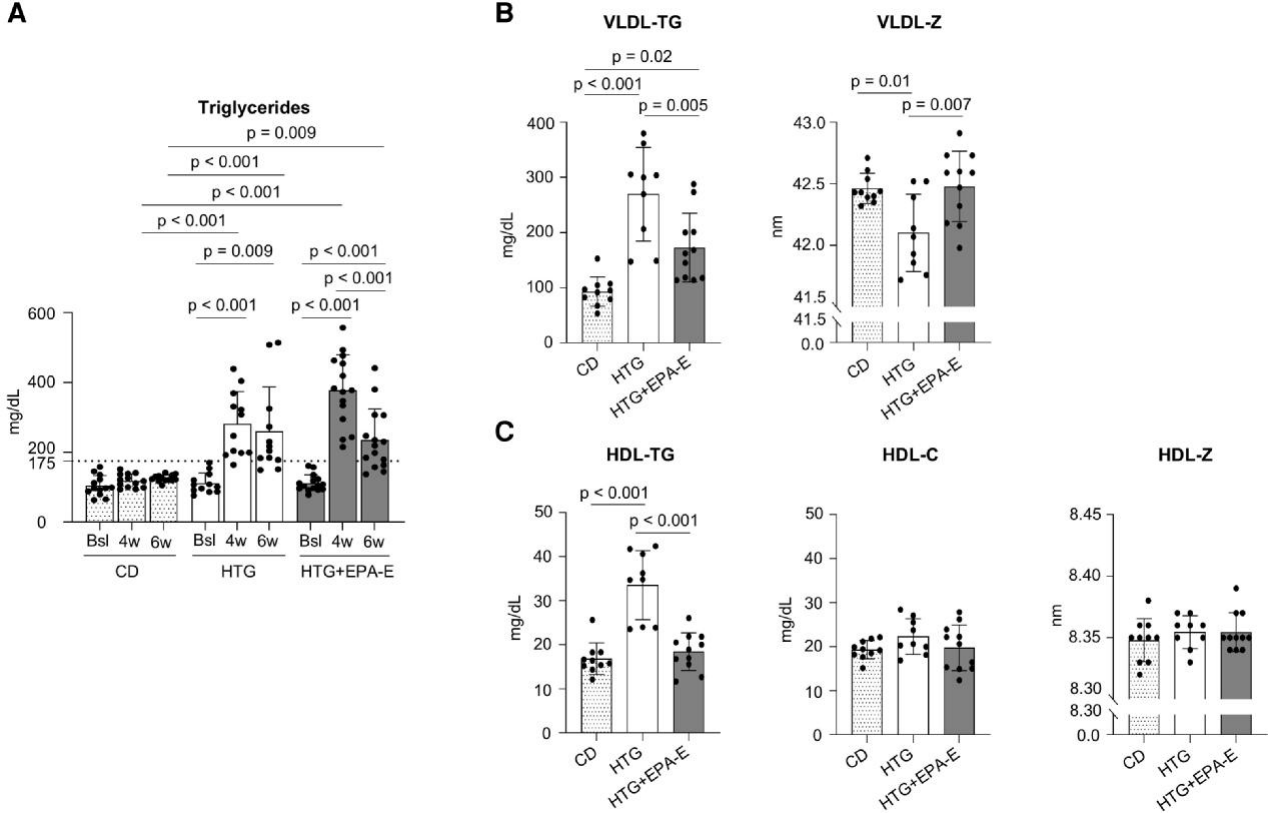
EPA-E effects in lipid parameters. (*A*) Serum triglyceride levels. (*B*) VLDL-TG levels and VLDL-Z at 6 weeks. (*C*) HDL-TG and HDL-C levels and HDL-Z at 6 weeks. CD, control diet group; HTG, hypertriglyceridaemia group; EPA-E, eicosapentaenoic acid ethyl ester-treated group; Bsl, basal; TG: triglycerides, VLDL: very low-density lipoprotein; HDL: high-density lipoprotein. Data are expressed as mean ± SD. *A*: *n* = 12–15 animals/group; *B* and *C*: *n* = 9–11 animals/group; *A*: mixed-effects model; *B* and *C*: one-way ANOVA; *C*_2_: Brown–Forsythe and Welch ANOVA

At 4 weeks, both the HTG and HTG + EPA-E groups showed a significant increase in serum TG levels (>175 mg·dL⁻¹) as compared to their baseline levels and the CD group. At 6 weeks, circulating TG levels remained unchanged in the HTG group, while significantly decreased in the HTG + EPA-E group (*Figure 2A*; *P* < 0.05 vs Week 4; Supplementary data online, *Figure S1*).

At 6 weeks, NMR spectrometry showed that VLDL triglycerides (VLDL-TG) levels were markedly increased in the HTG group. Eicosapentaenoic acid ethyl ester administration significantly reduced these levels (*P* < 0.05), although they remained higher than in the CD group. As per particle size, VLDL particles were smaller in the HTG group as compared to both CD and HTG + EPA-E groups (*Figure 2B*). HDL-TG levels increased only in the HTG group, and no changes in HDL cholesterol (HDL-C) levels and HDL particle size were detected among the different groups (*Figure 2C*). Total cholesterol decreased in all groups at 4 weeks but rose significantly only in the HTG group by Week 6 (see Supplementary data online, *Figure S2A*). Glucose levels remained within the physiological range throughout the entire experimental period in the animals fed with high-sucrose diet. In contrast, a time-dependent reduction in glucose concentrations was observed in the CD-fed group likely because of the high-alfalfa content in the diet.^33,34^ Finally, a significant increase in body weight was observed in all three groups throughout the study, without significant differences between the groups at week 6^35^ (see Supplementary data online, *Figure S2B*). Serum VLDL-C and RM-C levels did not differ among groups at 6 weeks (see Supplementary data online, *Figures S3A* and *S4A*).

In summary, EPA-E effectively reverses the atherogenic lipid profile and the VLDL changes induced by HTG.

### Effects of eicosapentaenoic acid ethyl ester on hypertriglyceridaemia-induced left ventricular contractile dysfunction and acute cardiac function following myocardial infarction

**Figure 3:**
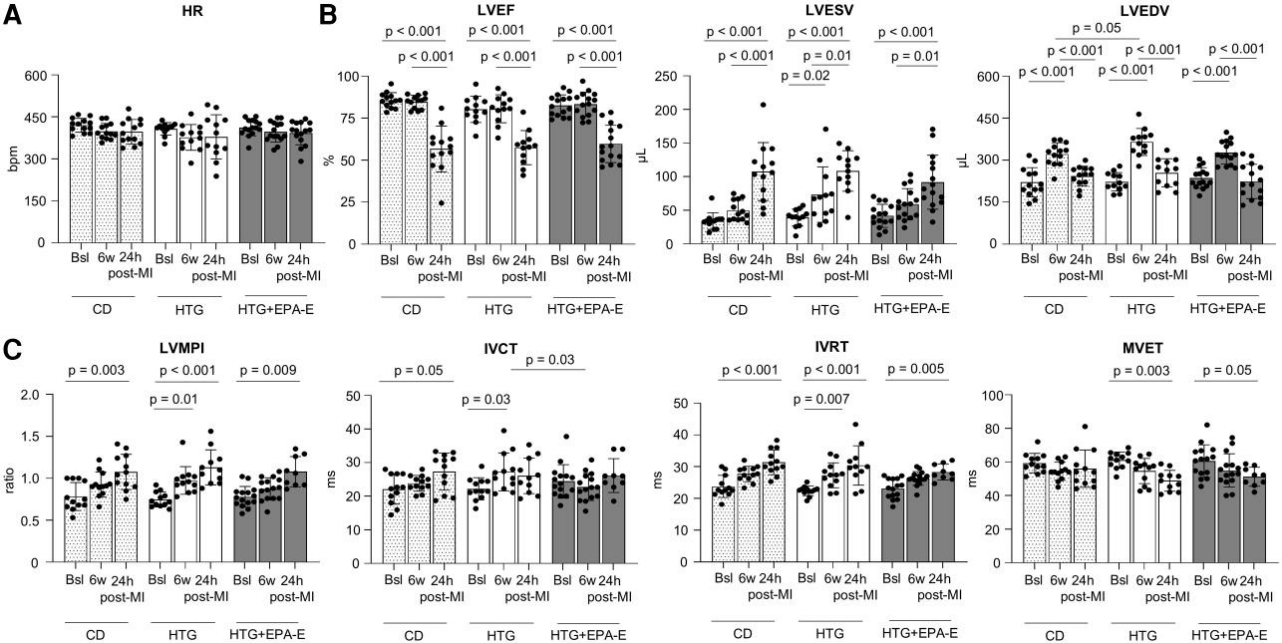
Echocardiographic studies. (*A*) Heart rate. (*B*) Left ventricle systolic-related parameters. (*C*) Left ventricular global function-related parameters. CD, control diet group; HTG, hypertriglyceridaemia group; EPA-E, eicosapentaenoic acid ethyl ester-treated group; MI, myocardial infarction; Bsl, basal; HR, heart rate; LVEF, left ventricular ejection fraction; LVESV, left ventricular end-systolic volume; LVEDV, left ventricular end-diastolic volume; LVMPI, left ventricular myocardial performance index; IVCT, isovolumic contraction time; IVRT, isovolumic relaxation time; MVET, mitral valve ejection time. Data are expressed as mean ± SD. *A* and *B*: *n* = 12–15 animals/group; *C*: *n* = 9–15 animals/group; *A*, *B*_1_, *B*_2_, *C*: mixed-effects model; *B*_3_: two-way ANOVA

No changes in HR were observed throughout the study and across the different animal groups (*Figure 3A*). As per cardiac function, although no differences were detected in the overall LVEF among the three groups, LVESV was found to be significantly impaired in the HTG group at 6 weeks. HTG + EPA-E prevented LVESV dysfunction and limited LEDV to levels comparable to that of the CD group (*Figure 3B*). At 6-week, a significant alteration in LVMPI was also observed in the HTG group as compared to the other two groups due to prolonged IVCT and IVRT. MVET time showed comparable variations among the different groups (*Figure 3C*).

Regarding cardiac function after MI, a comparable degree of myocardial deterioration was observed across the three animal groups assessed 24 h post-MI (*Figure 3A-C*). No differences were observed over the progression of LV Mass, LVAWs and LVAWd, MV *E/A*, LVSF, LVIDs, and LVIDd at 24 h post-MI among the groups (see Supplementary data online, *Figure S5A–D*).

In summary, EPA-E prevents HTG-induced left ventricular impairment and does not impact acute cardiac dysfunction in the setting of MI.

### Effects of eicosapentaenoic acid ethyl ester on survival during the ischaemic phase and acutely post-myocardial infarction

**Figure 4:**
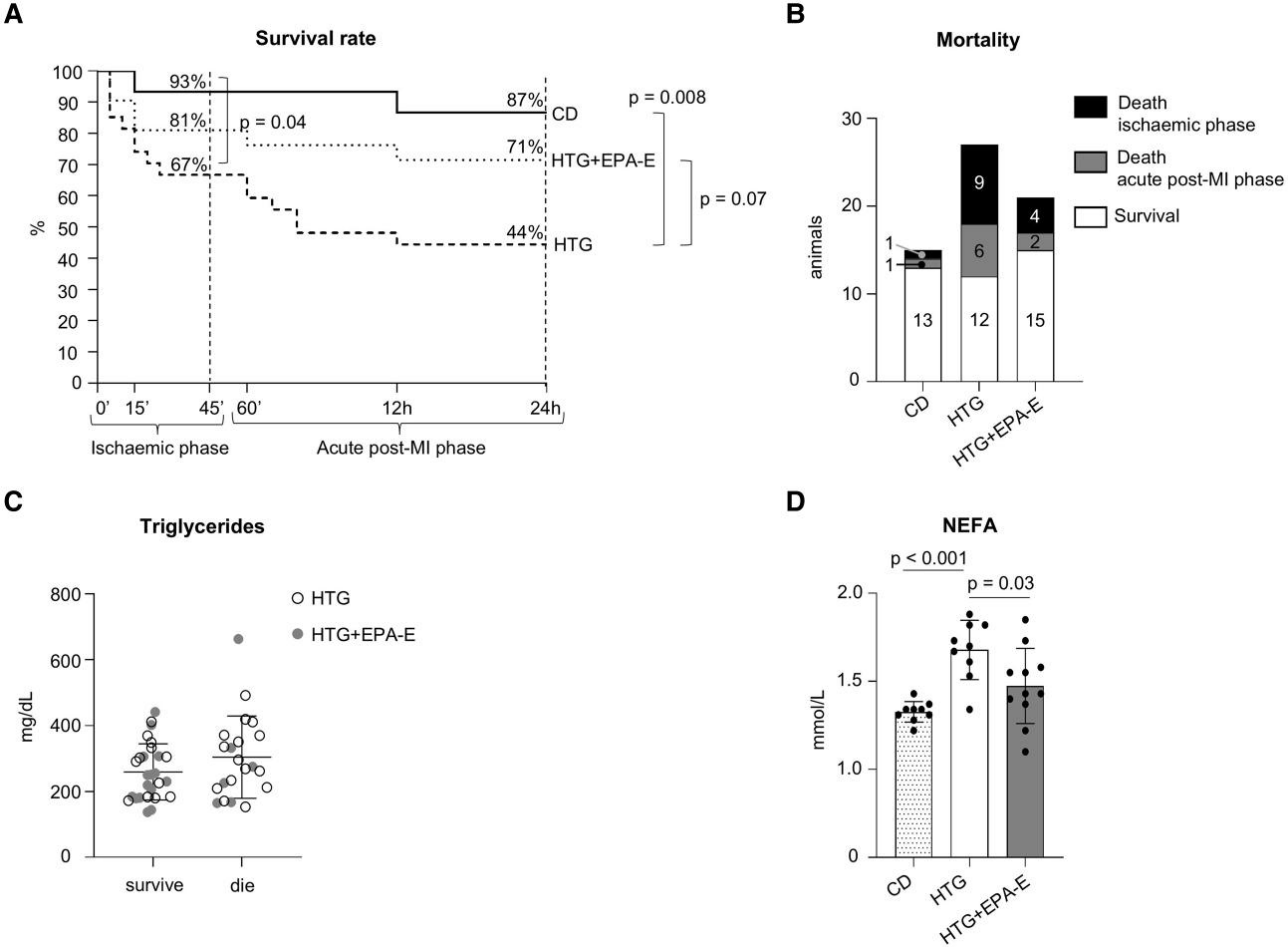
Impact of EPA-E on survival. (*A*) Kaplan–Meier curves during ischaemia (ischaemic phase) and acute post-MI phase. (*B*) Number of cases. (*C*) Serum TG levels at 6 weeks and mortality. (*D*) Circulating NEFA levels at 6 weeks. CD, control diet group; HTG, hypertriglyceridaemia group; EPA-E, eicosapentaenoic acid ethyl ester-treated group; MI, myocardial infarction; TG, triglycerides; NEFA, non-esterified fatty acids. Data are expressed as mean ± SD. *A*–*C*: *n* = 15–27 animals/group; *D*: *n* = 9–11 animals/group; *A*: log-rank (Mantel–Cox) test; *C*: Mann–Whitney test; *D*: one-way ANOVA

Kaplan–Meier curve revealed significantly lower survival rates in the HTG group (67%; *P* < 0.05) as compared to the CD group during the ischaemic phase. In contrast, the HTG + EPA-E group exhibited survival rates not statistically different from those of the CD group (93% vs 81%, respectively). The acute post-MI survival rate in the EPA-E group was comparable to that of the CD group. While survival improved in EPA-E-treated animals (71%) compared to the HTG group (44%), this difference did not reach statistical significance (*P* = 0.07; *Figure 4A*). Cox regression analysis performed during the ischaemic and acute post-MI phase indicated that HTG animals had a significantly higher risk of death compared with the CD group (HzR = 5.5; 95% CI = 1.26–24.18), whereas HzR of the HTG + EPA was of 2.14 (95% CI = 0.487–11.969) with no significant change vs CD group. Furthermore, when comparing EPA-E-treated rats to the untreated HTG group, EPA-E reduced the risk of death by 56% (HzR = 0.44; 95% CI = 0.17–1.14), suggesting a non-significant trend towards improved survival. As such, final mortality was 29% in EPA-E-treated animals representing nearly half of the 56% observed in non-treated HTG animals. In the CD group, the mortality rate was 13% (*Figure 4B*). No differences in serum TG levels at 6 weeks were detected between survivors and non-survivors in HTG or HTG + EPA-E groups (*Figure 4C*), indicating that circulating TG levels did not determine survival outcomes.

Circulating NEFA levels at 6 weeks were comparable between the CD and HTG + EPA-E groups, whereas they were found to be significantly higher in the HTG group (*Figure 4D*).

In summary, EPA-E shows a non-significant trend towards a reduction in mortality associated with MI independently of its TG-lowering effects.

### Effects eicosapentaenoic acid ethyl ester on infarct size

**Figure 5:**
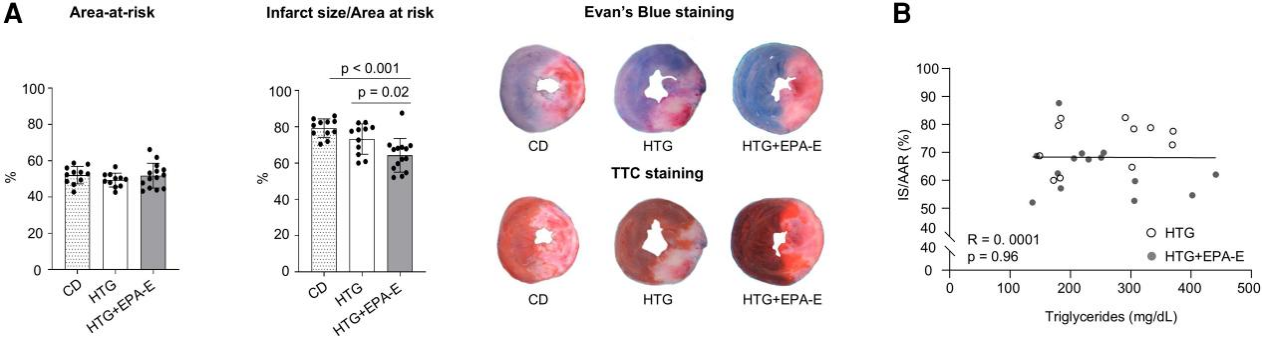
EPA-E effects on cardiac damage. (*A*) AAR and IS assessment. (*B*) Correlation between IS/AAR and circulating TG levels at 6 weeks. CD, control diet group; HTG, hypertriglyceridaemia group; EPA-E, eicosapentaenoic acid ethyl ester-treated group; AAR, area at risk; IS, infarct size; TTC, 2,3,5-triphenyltetrazolium chloride; TG, triglycerides. Data are expressed as mean ± SD. *A*: *n* = 11–14 animals/group; one-way ANOVA; *B*: linear regression

No differences in AAR were observed among all groups. HTG + EPA-E group showed significantly smaller infarcts as compared to both HTG and CD groups whereas no differences were found between CD and HTG animals (*Figure 5A*). No correlation was detected between IS and circulating TG levels in either the HTG or HTG + EPA-E animals (*Figure 5B*).

In summary, EPA-E significantly reduces IS independently of its hypotriglyceridaemic properties.

### Effects of eicosapentaenoic acid ethyl ester on apoptosis execution and fibrosis deposition

**Figure 6:**
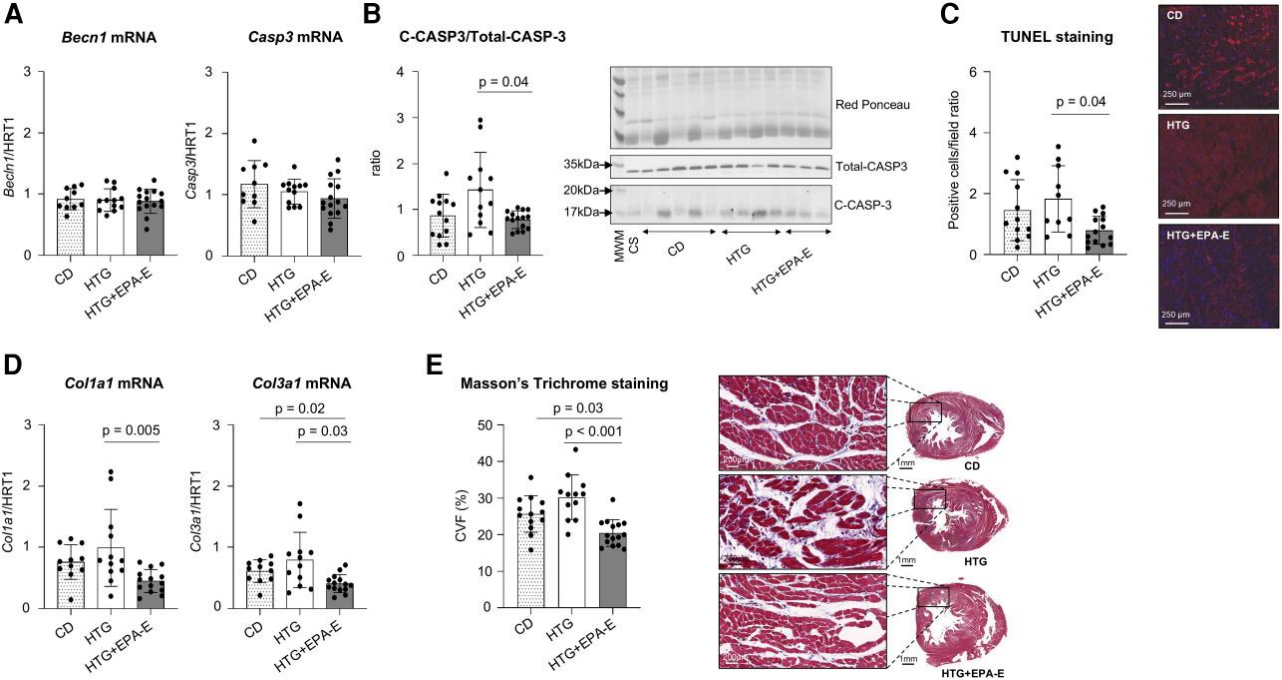
Impact of EPA-E on apoptosis execution and fibrosis in the infarcted heart. (*A*) *Becn1* and *Casp3* transcript levels. (*B*) Cleaved Caspase 3/Caspase 3 (C-CASP3/CASP3) ratio. (*C*) TUNEL staining. (*D*) *Col1a1 and Col3a1* transcript levels. (*E*) Masson’s trichrome staining. CD, control diet group; HTG, hypertriglyceridaemia group; EPA-E, eicosapentaenoic acid ethyl ester-treated group; CVF, collagen volume fraction. Data are expressed as mean ± SD. *A*: *n* = 10–15 animals/group; *B* and *E*: *n* = 12-15 animals/group; *C*: *n* = 10-14 animals/group; *D*: n = 11–14 animals/group. *A*, *D*_1_, *E*: one-way ANOVA; *B*, *C*, *D*_2_: Brown–Forsythe and Welch ANOVA; *C*: quantification in four random fields; scale bar 250 µm; *E*: quantification in five random fields; scale bar 200 µm and 1 mm

No differences were detected in the transcript levels of apoptotic markers *Becn1* and *Casp3* in the infarcted heart among all animals (*Figure 6A*). However, EPA-E treatment resulted in a lower C-CASPASE-3/Total-CASP3 ratio as compared with non-treated HTG animals (*Figure 6B*). TUNEL staining showed the same pattern, revealing lower apoptosis execution in the infarcted heart of EPA-E-treated animals vs the HTG group (*Figure 6C*).

Regarding cardiac fibrosis, administration of EPA-E was associated with a reduction in the transcript levels of *Col1a1* and *Col3a1* (*Figure 6D*), as compared to the HTG animals, and subsequent lower fibrillar collagen deposition assessed by Masson’s Trichrome staining (*Figure 6E*).

In summary, EPA-E attenuates post-MI injury by limiting cardiomyocyte apoptotic cell death and cardiac fibrosis deposition.

### Effects of eicosapentaenoic acid ethyl ester on the inflammatory response post-myocardial infarction

**Figure 7:**
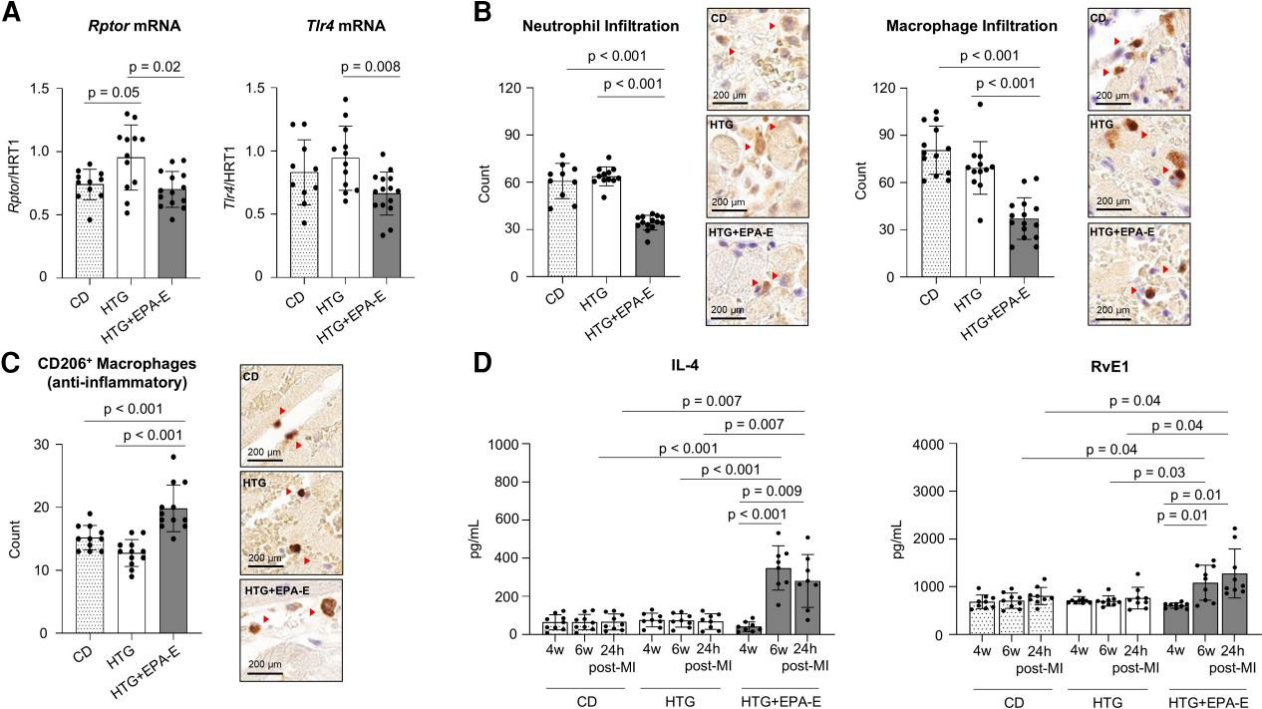
EPA-E local and systemic effects on the inflammatory response. (*A*) *Rptor* and *Tlr4* transcript levels in the infarcted heart. (*B*) Neutrophil and macrophage cardiac infiltration. (*C*) CD206^+^ macrophage detection in the infarcted heart. (*D*) Circulating IL-4 and RvE1 levels. CD, control diet group; HTG, hypertriglyceridaemia group; EPA-E, eicosapentaenoic acid ethyl ester-treated group; IL, interleukin; RvE1, resolvin E1. Data are expressed as mean ± SD. *A*_1_: *n* = 11–14 animals/group; *A*_2_: *n* = 10–15 animals/group; *B*_1_: *n* = 10–14 animals/group; *B*_2_: *n* = 12–15 animals/group; *B*_3_: *n* = 11–12 animals/group; *D*: *n* = 8–9 animals/group; *A*_2_, *B*_2_, *B*_3_, *C*: one-way ANOVA; *A*_1_, *B*_1_: Brown–Forsythe and Welch ANOVA; *D*: mixed-effects model; *B* and *C*: quantification in ten random fields; scale bar 200 µm

Eicosapentaenoic acid ethyl ester treatment was associated with a significant reduction in *Rptor* and *Tlr4* transcript levels in the infarcted heart compared to the HTG group, displaying a gene expression pattern that did not differ significantly from that of the CD group (*Figure 7A*).

Eicosapentaenoic acid ethyl ester treatment was associated with lower cardiac inflammatory cell recruitment, with neutrophil and macrophage counts decreasing by approximately 47% and 46%, respectively, compared to the other two groups (*Figure 7B*). Conversely, HTG + EPA-E animals displayed higher anti-inflammatory CD206^+^ macrophage content vs HTG and CD groups (*Figure 7C*). Of note, RM-C showed a positive correlation with CD206^+^ macrophage infiltration (*P* = 0.001, *R* = 0.35; Supplementary data online, *Figures S4G*). IL-4 and RvE1 circulating levels remained unchanged over time in the CD and HTG groups, whereas they were found to be significantly enhanced in HTG + EPA-E animals at 6-week and 24 h post-MI, as compared to pre-treatment levels (4 weeks) and to CD and HTG groups (*Figure 7D*).

In summary, EPA-E modulates the post-MI immune response by reducing inflammation and promoting a pro-resolving response.

### Effect of eicosapentaenoic acid ethyl ester on mitochondria, oxidative stress, and AKT in the infarcted heart

**Figure 8:**
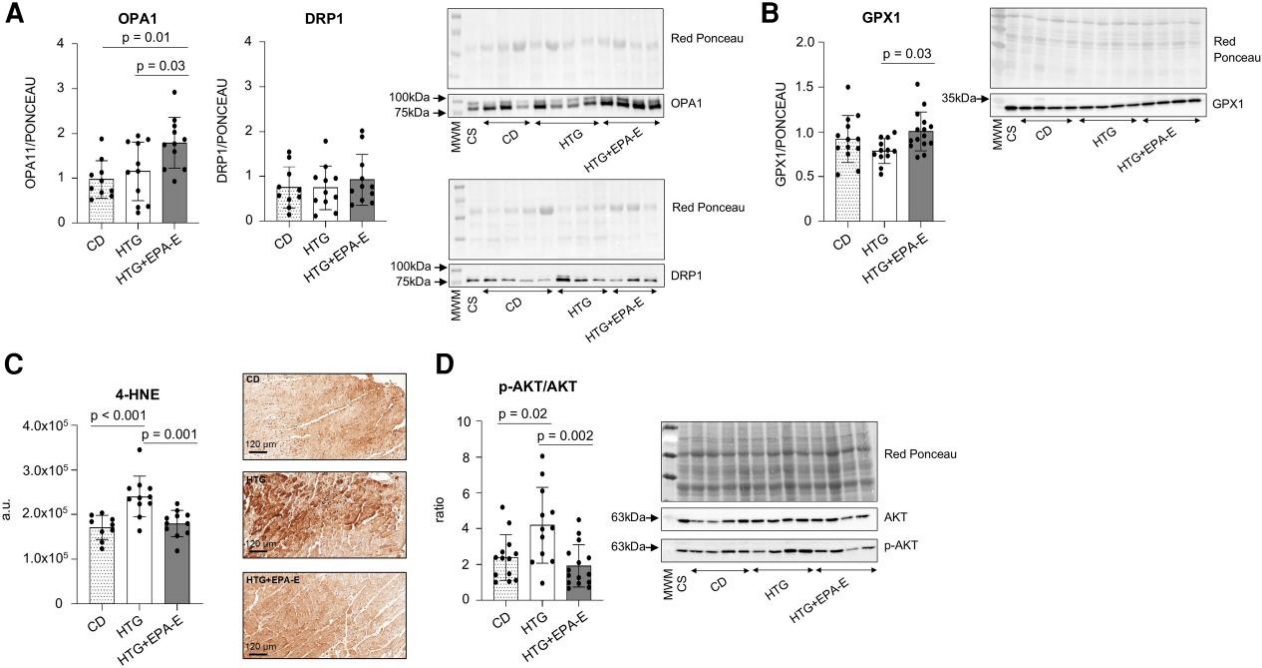
Impact of EPA-E in mitochondrial dynamics and AKT-mediated oxidative stress in the infarcted heart. (*A*) OPA1 and DRP1 cardiac mitochondrial protein levels. (*B*) GPX1 protein expression in the infarcted heart. (*C*) 4-HNE levels in the infarcted heart. (*D*) p-AKT/AKT ratio in the infarcted heart. CD, control diet group; HTG, hypertriglyceridaemia group; EPA-E, eicosapentaenoic acid ethyl ester-treated group; OPA1, optic atrophy protein 1; DRP1, dynamin-related protein 1; GPX1, glutathione peroxidase 1; 4-HNE, 4-hydroxynonenal; AKT, protein kinase B (PKB); a.u., arbitrary units. Data are expressed as mean ± SD. *A*: *n* = 10–11 animals/group; *B* and *D*: *n* = 12-15 animals/group; *C*: *n* = 9–11 animals/group; *A*–*D*: one-way ANOVA; *C*: quantification in ten random fields; scale bar 120 µm

We assessed mitochondrial proteins in the infarcted myocardium and found increased OPA1 levels in the EPA-E group as compared to CD and HTG animals, while DRP1 levels remained unchanged among all groups (*Figure 8A*). Eicosapentaenoic acid ethyl ester–treated animals showed significantly higher GPX1 levels and lower 4-HNE levels as compared to the HTG group (*Figure 8B* and *C*). No differences were observed between EPA-E-treated and CD animals. An enhanced p-AKT/AKT ratio was also observed in the HTG group as compared to EPA-E and CD groups, the latter two showing a comparable degree of activation rates (*Figure 8D*).

In summary, EPA-E preserves mitochondrial integrity, mitigates oxidative damage, and restores AKT signalling in the infarcted heart.

### Effects of eicosapentaenoic acid ethyl ester on the cardiac lipidomic and metabolic signatures

**Figure 9:**
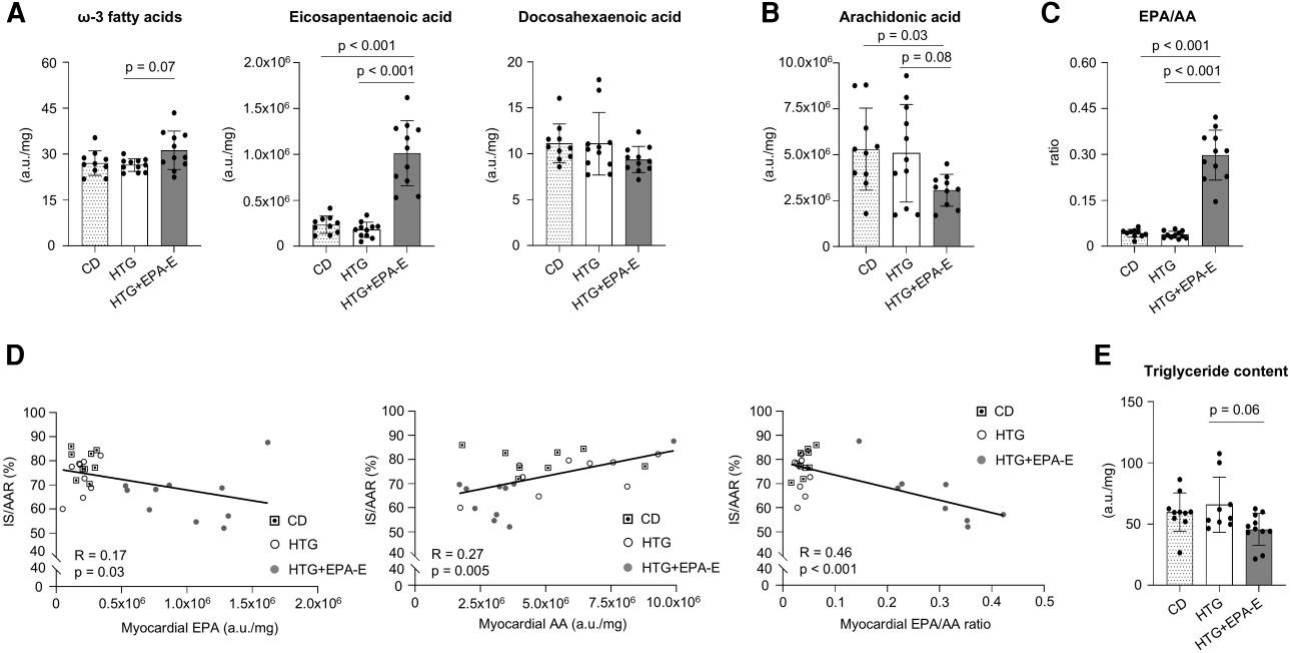
Heart lipidomics. (*A*) ω-3 fatty acids, EPA, and DHA levels. (*B*) AA levels. (*C*) EPA/AA ratio. (*D*) Correlation between IS and EPA levels (right), AA levels (middle), and the EPA/AA ratio (left). (*E*) Cardiac triglycerides content. CD, control diet group; HTG, hypertriglyceridaemia group; EPA-E, eicosapentaenoic acid ethyl ester-treated group; DHA, docosahexaenoic acid; AA, arachidonic acid; a.u., arbitrary units. Data are expressed as mean ± SD. *A*–*C*: *n* = 10–11 animals/group; *E*: *n* = 9–11 animals/group; *A*_1_, *A*_2_, *B*, and *C*: Brown–Forsythe and Welch ANOVA; *A*_3_ and *E*: Kruskal–Wallis; *D*: linear regression

Targeted lipidomic analysis revealed a non-significant increase in ω-3 fatty acid levels in the infarcted hearts of EPA-E-treated animals as compared to the other two groups (*P* = 0.07 vs HTG). Eicosapentaenoic acid was markedly increased in HTG-EPA-E-treated animals whereas no differences were detected in docosahexaenoic acid (DHA) levels among all groups (*Figure 9A*). Animals treated with EPA-E showed significantly lower levels of AA compared to CD animals (*P* = 0.03). However, the differences between the EPA-E and HTG groups were not statistically significant (*P* = 0.08) (*Figure 9B*), leading to a significantly higher EPA/AA ratio (*Figure 9C*). An inverse correlation was identified between IS and cardiac EPA levels, while a direct correlation was observed between IS and AA levels. However, the strongest association was found between IS and the EPA/AA ratio (*Figure 9D*). Finally, intracardiac TG content showed a trend to lower values in EPA-E-treated animals compared to the HTG group; however, this difference did not reach statistically significance (*P* = 0.06) (*Figure 9E*).

**Figure 10:**
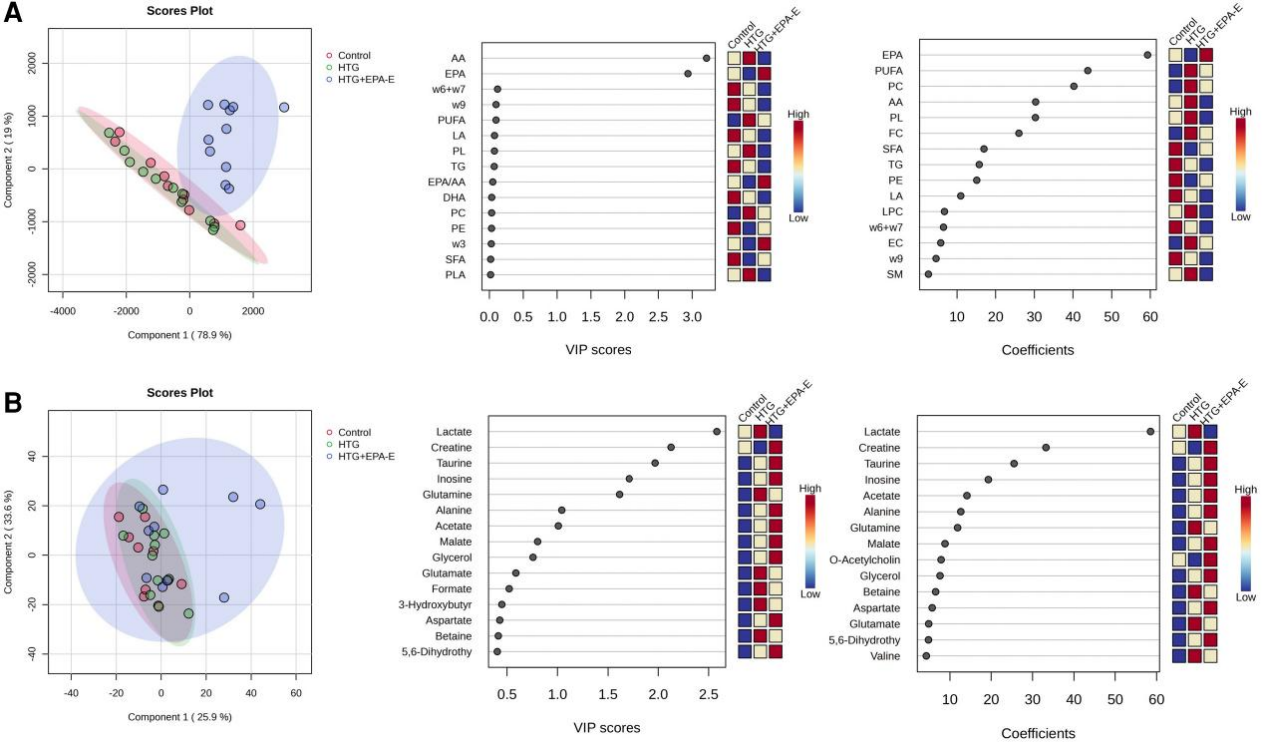
Multivariate PLS-DA analysis of the infarcted myocardium. (*A*) Lipidomic profile. (*B*) Metabolomic profile. Score scatter plot based on the PLS-DA models (red for CD; green for HTG and blue for HTG + EPA-E groups). Rank of the different lipids and metabolites (the top 15) identified by the PLS-DA according to the VIP and coefficient scores on the X-axis. The colour boxes indicate whether metabolite concentration is high (red), intermediate (ochre), or low (blue). CD, control diet group; HTG, hypertriglyceridaemia group; EPA-E, eicosapentaenoic acid ethyl ester-treated group; AA, arachidonic acid. *A* and *B*: *n* = 10–11 animals/group; 1000-fold permutation tests and 5-fold cross-validation approach

Multivariable PLS-DA of the lipid profile obtained from the ^1^H-NMR analysis in the infarcted heart revealed a distinct cluster separation between the EPA-E-treated animals and both the HTG and CD groups underscoring the significant differences in lipid profiles associated with EPA-E treatment. The first two components explained 97.9% of the variance (C1: 78.9%; C2: 19%), with strong model performance (accuracy = 0.69, *R*² = 0.66, *Q*² = 0.59), and high statistical significance (permutation test *P* < 0.001). Major contributing lipids included EPA and AA (high VIP scores), as well as polyunsaturated fatty acids (PUFA), phosphatidylcholine (PC), glycerophospholipids (PL), and free cholesterol (FC), which exhibited high coefficient values (*Figure 10A*), indicating significant EPA-E-mediated remodelling of myocardial lipid composition.

As per myocardial metabolomics of the infarcted heart, PLS-DA revealed overlapping clustering among all three groups (Component 1: 25.9%; Component 2: 33.6%), with poor model performance (accuracy = 0.49, *R*² = 0.25, *Q*² = 0.1) and no statistical significance (*P* = 0.47). Despite this, VIP and coefficient analyses revealed metabolic shifts in the EPA-E group relative to HTG, driven by lactate, creatine, taurine, inosine, and glutamine (*Figure 10B*).

In summary, EPA-E improves lipidomic and metabolic profiles in the infarcted heart.

### Effect of eicosapentaenoic acid ethyl ester on serum lipidomic and metabolic profiles

**Figure 11:**
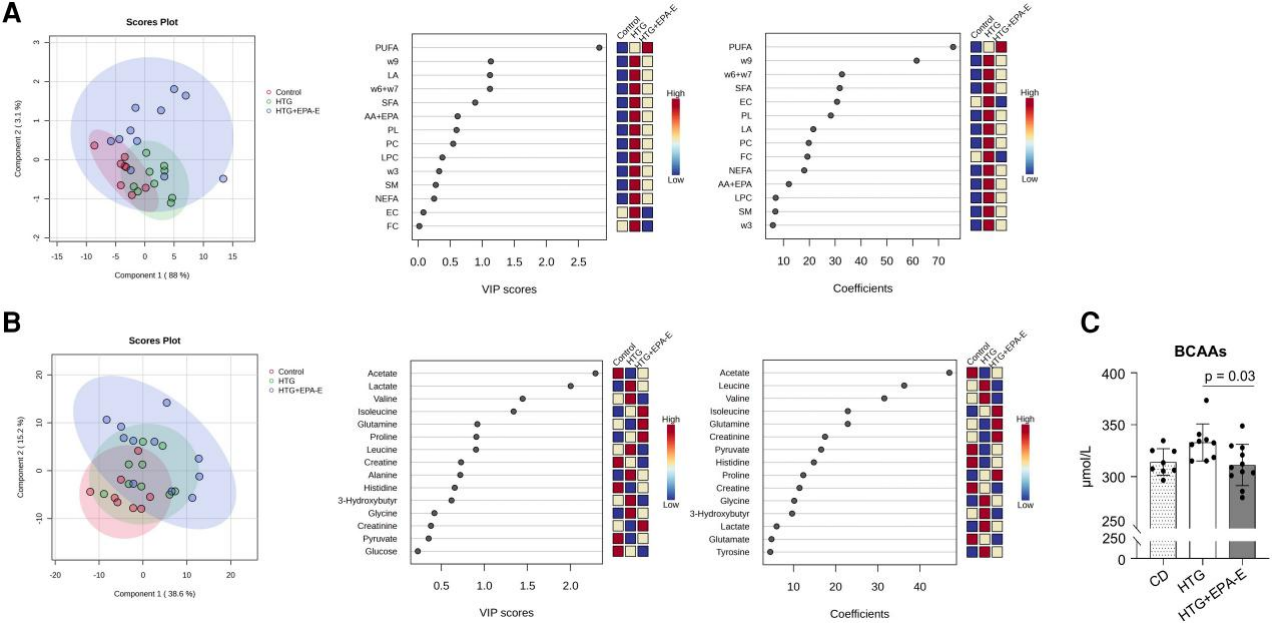
Multivariate PLS-DA analysis of the serum at 6 weeks. (*A*) Lipidomic profile. (*B*) Metabolomic profile. Score scatter plot based on the PLS-DA models (red for CD, green for HTG, and blue for HTG + EPA-E groups). Rank of the different lipids and metabolites (the top 15) identified by the PLS-DA according to the VIP and coefficient scores on the X-axis. The colour boxes indicate whether metabolite concentration is high (red), intermediate (ochre), or low (blue). CD, control diet group; HTG, hypertriglyceridaemia group; EPA-E, eicosapentaenoic acid ethyl ester-treated group; BCAAs, branched chain amino-acids. *A*–*C*: n = 8–11 animals/group; *A* and *B*: 1000-fold permutation tests and 5-fold cross-validation approach; *C*: one-way ANOVA

Multivariate PLS-DA of the serum lipid profile obtained from ^1^H-NMR analyses at 6 weeks also revealed a clear distinct clustering pattern that distinguished EPA-E-treated rats from both HTG and CD groups. The first two components explained 91.1% of the variance (C1: 88%; C2: 3.1%), with strong model performance (accuracy = 0.72, *R*² = 0.55, *Q*² = 0.28) and strong statistical significance (permutation test *P* = 0.005). Major contributing lipids included PUFA, ω-9 fatty acids, linolenic acid (LA), ω-6-ω-7 fatty acids, and saturated fatty acids (SFA), as reflected by their high VIP scores and coefficient values (*Figure 11A*).

Regarding the serum metabolomic profile, PLS-DA revealed partial overlap among the three experimental groups (Component 1: 38.6%; Component 2: 15.2%), accompanied by limited model performance (accuracy = 0.35, R² = 0.70, Q² = 0.10) and modest statistical significance (*P* = 0.023). Nevertheless, VIP and coefficient analyses identified distinct metabolic shifts in the EPA-E group compared with HTG, primarily driven by acetate, lactate, valine, and isoleucine (high VIP scores), as well as leucine and glutamine (high coefficient values) (*Figure 11B*). Notably, EPA-E treatment resulted in a significant reduction in circulating branched-chain amino acids (BCAAs; leucine + isoleucine + valine) compared with the HTG group (*Figure 11C*). VLDL-C showed a significant positive correlation with circulating BCAAs (*P* = 0.005, *R* = 0.26); Supplementary data online, *Figures S3I*).

In summary, EPA-E reduces circulating BCAAs, potentially linking lipid modulation to metabolic improvement.

## Discussion

This study, conducted in an experimental model of HTG and MI, demonstrates that EPA-E administration reduces IS independent of changes in circulating TG. These direct cardioprotective benefits result from an enhanced pro-resolving inflammatory response, both systemically and locally within the heart, as well as reduced apoptosis, fibrosis, oxidative stress, and lipotoxicity and less mitochondrial dysfunction in the infarcted heart. Additionally, EPA-E treatment improves overall serum and cardiac lipidomic and metabolomic profiles, likely enhancing cardiac resilience to MI.

First, we demonstrate that EPA-E treatment exerts cardioprotection^36^ and limits IS. We have previously demonstrated that hypercholesterolaemia is associated with larger infarcts.^37^ Here, we further explore the impact of another type of lipidic dysregulation, increased TG levels, on myocardial function and IS. In contrast to the setting of elevated LDL-C, high serum TG levels were not associated with larger infarcts than in CD. Similarly, the innate immune response triggered in the infarcted region was not changed by HTG. However, EPA-E-treated animals developed significantly smaller infarcts, an effect that was not associated with changes in TG levels. In addition, we also observed that EPA-E treatment was associated with a 17.3% reduction in MI-related mortality during the ischaemic phase and a 38% decrease during the acute post-MI phase (both compared to the HTG group), although it did not reach significance. This mortality decline may be associated with a reduction in fatal arrhythmias. In this regard, large cohort trials^38,39^ and experimental studies^40^ have supported a link between circulating NEFA levels and ventricular arrhythmias and sudden cardiac death after MI.^39^ We detect higher circulating NEFA levels in the HTG group as compared to EPA-E-treated animals. Besides, preclinical studies suggest that EPA-E’s exert antiarrhythmic effects^41,42^ by modulating ion channels and reducing fibrosis; yet, clinical evidence remains inconclusive.^43^ Interestingly, despite earlier reports indicating a positive correlation between circulating TG levels and the development of atrial fibrillation,^44^ Eicosapentaenoic acid ethyl ester potential pro-survival benefits appeared to be independent of serum TG levels.

As per the potential mechanisms of cardioprotection, excessive TG accumulation in the myocardium has been associated with lipotoxicity, resulting in impaired cardiac function.^45^ Hypertriglyceridaemia animals displayed elevated intracardiac TG content and developed cardiac dysfunction at 6 weeks, as evidenced by increased LVESV^46^ and LVMPI,^47^ a parameter shown to be more sensitive than LVEF.^48^ Conversely, EPA-E treatment mitigated cardiac lipotoxicity, thereby preventing cardiac function impairment. These findings build upon recent evidence demonstrating EPA’s ability to reduce renal lipotoxicity,^49^ highlighting its broader protective effects against lipid-induced organ damage. Eicosapentaenoic acid ethyl ester administration may have also contributed to preserve cardiac function by lowering cardiac AA levels. AA has been shown to act as a lipid messenger and activate the cardiac RhoA/ROCK pathway,^50^ a signalling cascade known to promote cardiomyocyte apoptosis and the development of heart failure.^51^ Although EPA-E prevented HTG-induced cardiac dysfunction it did not attenuate cardiac impairment during the acute phase post-MI. However, the beneficial effects of EPA-E on cardiac performance have shown to become evident over the longer-term recovery period following MI.^52^

We also detected reduced *Tlr4* and *Rptor* transcript levels in the infarcted hearts of EPA-E-treated animals, indicating an attenuation of the pro-inflammatory response. TLR4 is a key pattern recognition receptor mainly involved in the cardiac innate immune response^53^ and the mTOR pathway, encoded by the *Rptor* gene, serves as a downstream mediator of this pro-inflammatory response,^54^ contributing to impaired autophagic clearance of damaged cellular components.^53,55^ Supporting these molecular findings, the infarcted heart of the HTG + EPA-E group showed decreased recruitment of pro-inflammatory cells and a shift towards a higher abundance of anti-inflammatory CD206⁺ macrophages in concurrence with higher systemic IL-4 levels as compared to the other two groups. EPA-E treatment has been shown to modulate immune responses by reducing pro-inflammatory Th1 cells and promoting Th2 cells, which secrete IL-4.^25^ IL-4, in turn, has been shown to support tissue regeneration,^56^ shift monocyte polarization towards an anti-inflammatory phenotype,^57^ and limit neutrophil cardiac migration by regulating endothelial adhesion molecules^58^ further supporting our findings. On the other hand, EPA competes with AA as a substrate for cyclooxygenase and lipoxygenase pathways,^59^ promoting the production of pro-resolving mediators such as resolvins (RvE1 and RvE2),^60^ as detected in EPA-E-treated animals. These overall anti-inflammatory effect and pro-resolving state likely explains the reduction in cardiac fibrosis and improved cardiac repair observed in EPA-E-treated animals post-MI.^61^ Experimental studies have shown that long-term treatment with EPA-E (1 g/day)^26^ exerts anti-fibrotic effects by blocking the phospho-Smad2/3 from TGF-β pathway.^26,27^ We evidence that a short-term EPA-E treatment suffices to downregulate *Col1a1* and *Col3a1* gene expression and subsequent collagen I/III fibre deposition in the infarcted heart. Additionally, previous *in vitro* studies in H9C2 cardiomyocytes have revealed that EPA-E treatment modulates autophagy and limits apoptosis execution.^62^ We extend these observations by providing *in vivo* evidence of EPA-E’s-related anti-apoptotic effects in the context of MI.

We also detected mitochondrial and antioxidant protectives effect of EPA-E in the setting of MI. EPA-E increased OPA1 levels in the infarcted myocardium, supporting mitochondrial fusion and cristae integrity,^63^ mechanisms known to reduce apoptosis and reactive oxygen species (ROS) generation.^64^ Likewise, EPA-E preserved GPX1 expression and markedly decreased 4-HNE accumulation,^65^ indicating reduced lipid peroxidation and oxidative stress.^66^ Besides, EPA-E prevented the AKT overactivation observed in HTG animals. Excessive AKT activation is indicative of a maladaptive response linked to oxidative stress, inflammation, apoptosis,^67,68^ and worse MI outcomes.^69^

Eicosapentaenoic acid ethyl ester treatment promoted a more resilient metabolic phenotype after MI, as reflected by reduced myocardial lactate levels, an early marker of diminished anaerobic burden.^70^ Additionally, elevated taurine levels enhance Na⁺ and Ca²⁺ handling and exhibit anti-apoptotic effects,^71^ while increased creatine contributes to ATP buffering and pH stability during ischaemic conditions.^72^ Moreover, elevated inosine levels provide complementary anti-inflammatory^73^ and antioxidant benefits.^74^ Furthermore, the observed shift in glutamine metabolism (characterized by lower glutamine and higher alanine) indicates improved energy homeostasis and aligns with IL-4-mediated anti-inflammatory cardiac remodelling.^75,76^

In addition to the changes in EPA and AA levels, EPA-E treatment modulated other lipid species relevant to MI pathology. During MI, the observed reduction in PC and lysophosphatidylcholine (LPC) levels indicates decreased ROS-mediated membrane peroxidation and an attenuated pro-inflammatory chemotactic signalling.^77,78^ Lower levels of PUFA may further mitigate oxidative damage to membrane lipids, particularly in the context of metabolic stress.^79^ Moreover, by replacing AA in membrane phospholipids, EPA helps to diminish FC accumulation, enhances membrane fluidity, and attenuates inflammation and endothelial dysfunction.^80^

Eicosapentaenoic acid ethyl ester treatment also improved the serum metabolic profile at 6 weeks, primarily by reducing circulating BCAAs (especially leucine and valine). Elevated BCAAs are strongly associated to CVD^81^ and can activate AKT/mTOR signalling, promoting mitochondrial ROS generation^82^ and contributing to pro-arrhythmogenic and pro-inflammatory effects.^83^ Conversely, the increased glutamine levels in the EPA-E group may enhance antioxidant protection and anti-inflammatory pathways through mechanisms involving HO-1 induction and improved glutathione metabolism.^84^ EPA-E-treated animals also exhibited elevated acetate concentrations, which are associated with reduced inflammation, oxidative stress, and apoptosis.^85^ Finally, the observed lactate-TG association suggests enhanced utilization of TG-derived substrates in EPA-E-treated animals.^86,87^

Moreover, the increase in PUFA levels in EPA-E-treated animals may reflect increased systemic EPA availability, consistent with its higher myocardial content, whereas lower circulating SFA is compatible with decreased VLDL-TG secretion^88^ and reduced activation of TLR4/NF-κB inflammatory pathways.^89^ In contrast, ω-7 and ω-9 fatty acids have been linked to higher all-cause mortality^90^ and to vascular smooth muscle cell dysfunction,^91^ respectively.

Finally, consistent with clinical findings, we report that EPA-E treatment reduced circulating TG levels by approximately 35%, improved the TRLs profile and VLDL particle diameter, key features of atherogenic dyslipidaemia.^21,22^ Besides, we observed a positive correlation between BCAAs and VLDL-C, consistent with their association with atherogenic lipid profiles,^92^ whereas the expected pro-inflammatory association between RM-C and immune infiltration^93^ was attenuated, likely reflecting EPA-E’s anti-inflammatory potential.^94^ Of note, although rats lack CETP,^95^ EPA-E can modulate lipid profiles via CETP-independent pathways, including increased lipoprotein lipase^96^ and hepatic/endothelial lipase activity.^97^

Our work has certain limitations that deserve to be acknowledged. First, we did not evaluate the impact of sex, as female rats are typically resistant to the development of diet-induced HTG condition.^35^ Additionally, our study focused on the acute effects of EPA pre-treatment on cardiac damage. Further studies are needed to determine whether these short-term benefits translate into reduced adverse cardiac remodelling and improved cardiac function over longer follow-up periods. Finally, including a group treated with EPA-E + statins would improve comparability with the REDUCE-IT trial. However, the following study was designed to isolate the effects of EPA-E beyond TG reduction, using an animal model that demonstrated no changes in other lipid parameters, particularly LDL-C, which is the primary target of statin therapy.

## Conclusion

Our study demonstrates that 2 weeks of EPA-E supplementation effectively reduces circulating TG levels, enhances the overall lipid profile, and ameliorates cardiac dysfunction associated with HTG. Most notably, EPA-E pre-treatment limits IS independent of its serum TG-lowering effect. These cardioprotective benefits result from an attenuated local and systemic inflammatory response, diminished apoptotic execution, reduced oxidative damage, lower fibrosis, attenuated mitochondrial dysfunction, and protection of lipidomic and metabolomic signatures that confer cardiac resilience to MI (*Structured Graphical Abstract*).

## Supplementary Material

ehag187_Supplementary_Data

## Declarations

### Data Availability

All data generated or analysed during this study are included in this published article (and its supplementary information files).
