# Supplementary material for: Eicosapentaenoic acid ethyl ester, cardiac metabolomic and lipidomic signatures, and cardioprotection in myocardial infarction
Source: Eur Heart J. 2026 Apr 17;47(31):4307–23. doi: 10.1093/eurheartj/ehag187 (PMC13472692; doi:10.1093/eurheartj/ehag187)
Supplement: Supplementary file 1 [file ehag187_supplementary_data.zip › Alcover et al_Supp_ Material_EURHEARTJ-D-25-03216-R2.docx]

**Eicosapentaenoic acid ethyl ester exerts cardioprotection and improves cardiac metabolomic and lipidomic signature in the setting of myocardial infarction**

Sebastià Alcover ^1,2^, Lisaidy Ramos-Regalado ^1,2^, Natàlia Muñoz-García ^1,2^, Gabriela O. Girón ^1,3^, Sergi Otero^1,3^, Sergi López ^1^, Teresa Padro^1,4^,

Lina Badimon ^1,4,5^, Gemma Vilahur ^1,4^

^1^ Institut de Recerca Sant Pau (IR SANT PAU), 08041 Barcelona, Spain.

^2^ Facultat de Biologia, Universitat de Barcelona, 08028, Barcelona, Spain.

^3^ Facultat de Biociències, Universitat Autònoma de Barcelona, 08193, Barcelona, Spain.

^4^ Centro de Investigación Biomédica en Red Enfermedades Cardiovasculares Instituto de Salud Carlos III, 28029, Madrid, Spain.

^5^ Facultat de Medicina, Universitat de VIC-UCC and Cardiovascular Research Foundation for Health Prevention and Innovation (FICSI), 08017 Barcelona, Spain.

Sebastià Alcover (0000-0003-3699-1769)

Lisaidy Ramos-Regalado (0009-0004-3512-8474)

Natàlia Muñoz-García (0000-0002-2982-8982)

Gabriela O. Girón (0009-0009-4753-0985)

Sergi Otero (0000-0002-2038-6882)

Sergi López (0000-0003-1794-879X)

Teresa Padró (0000-0003-1921-954X)

Lina Badimon (0000-0002-9162-2459)

Gemma Vilahur (0000-0002-2828-8873)

**Correspondence to:**

**Gemma Vilahur**

Director Cardiovascular Disease Area - *Sant Pau Research Institute (IR SANT PAU), Centre CERCA - CIBERCV*

*C/ Sant Quintí 77-79, 08041 Barcelona*

*Tel: 00 34 93 5537100*

e-mail: [gvilahur@santpau.cat](mailto:gvilahur@santpau.cat)

**Supplemental Material**

**METHODS**

The study design was reviewed and approved by the Institutional Animal Care and Use Committees (CEEA-IR, procedure #152) and authorised by the Animal Experimental Committee of the local government by the Spanish law (RD 53/2013) and European Directive 2010/63/EU. This study complies with the Guide for the Care and Use of Laboratory Animals published by the USA National Institutes of Health (NIH Publication No. 85-23, revised 1985), adheres to the ARRIVE guidelines 2.0 (1), and follows the *Practical Guidelines for Rigor and Reproducibility in Preclinical and Clinical Studies on Cardioprotection* (2,3). Furthermore, we are committed to the "3Rs" principle and ensured the use of the minimum number of animals necessary to achieve statistical significance.

**Experimental Design**

The experimental study design is detailed in **Figure 1**. Male Wistar Hannover rats (n = 63, weighing 125-150g, four weeks old; Janvier Laboratory) were housed in standard cages (two/cage) and maintained under control temperature (24ºC ±1) and humidity (55% ±5), with a 12h light-dark cycle. The animals had *ad libitum* access to water. All animals were allowed to acclimatise for five days and were fed a regular chow *ad libitum* before we conducted all baseline measurements (Teklad Global 14% Protein Rodent Maintenance Diet, Envigo). All animals were randomly allocated in the three experimental groups before baseline measurements: 1) a control diet group (CD; n = 15; 28.4% alfalfa, 6.4% sugars; R8960A01R, Safe); 2) a high-sucrose diet group (HTG; n = 27; 2.8% alfalfa, 56.2% sugars; U8960A01R, Safe) and 3) a high-sucrose diet EPA-E-treated group (HTG+EPA-E; n = 21; 2.8% alfalfa, 56.2% sugars; U8960A01R, Safe). The randomisation was performed using Excel software protocol, as follows: Animal IDs were listed in column A (A2:A64). A random number was generated in column B using the formula =RAND() for each animal. The list was then sorted ascending by column B to randomise the order, and animals were sequentially assigned to groups: All animals had free access to food and water *ad libitum* throughout the entire study period. Diets compositions are detailed in **Supplemental Table 1**. This process was performed once by SL blinded to group identities. The randomisation sheet is available upon request.

HTG was confirmed at four weeks based on fasting TG levels of >175 mg·dL^-1^, according to the most recent European guidelines (4) and the TG threshold used to stratify patients in clinical trials (5). In addition, the high-sucrose-fed animals exhibited a 2.8-fold increase in TG relative to the CD group, a magnitude of elevation that closely mirrors that described in the hereditary HTG rat model (6). After the assessment of HTG condition, HTG-EPA-E received a daily oral gavage dose of 0.3 g·kg⁻¹ of EPA-E, (99% purity, Amarin Pharma Inc., NJ, USA), for two weeks, and the HTG group received an equivalent volume of oral gavage drinking water. The dose of EPA-E was selected according to the 4 g/day dose in humans after adjusting for BSA (7). Thereafter, MI was induced through transient ligation of the left anterior descending coronary artery (LAD) for 45min. All animals were sacrificed 24h post-MI for IS assessment and tissue and blood sample collection. Sample labelling was carried out with alphanumeric codes to ensure blinding throughout all molecular, histological, and analytical assessments. Serial echocardiographic analyses were performed in all animals at baseline, at six weeks and 24h post-MI. The stated analyses were performed exclusively on samples or measurements obtained from animals that survived the entire experimental procedure. Variations in sample size across the different analyses are detailed in **Supplemental Table 2**.

**Transthoracic echocardiography**

All animals underwent transthoracic echocardiography to accurately assess cardiac function according to the indications provided by *the ESC Working Group on Myocardial Function* (8) and following our previous studies (9) by using a linear-array transducer system (VEVO F2; VisualSonics, Amsterdam, Netherlands). We assessed left ventricle (LV) ejection fraction (LVEF), LV shortening fraction (LVSF), LV anterior wall thickness in systole (LVAWs), LV anterior wall thickness in diastole (LVAWd), LV internal diameter in systole (LVIDs), LV internal diameter in diastole (LVIDd), LV posterior wall thickness in systole (LVPWs), LV posterior wall thickness in diastole (LVPWd), LV mass, LV end-systolic volume (LVESV) and LV end-diastolic volume (LVEDV) (n = 12-15 animals/group; **Supplemental Table 2**). Diastolic dysfunction was determined by assessing the following parameters from mitral inflow Doppler images: isovolumic contractile time (IVCT), isovolumic relaxation time (IVRT), mitral valve ejection time (MVET) and *E* wave/*A* wave (*E/A* ratio). Finally, LV myocardial performance index (LVMPI) was calculated to evaluate overall cardiac function. Three representative cardiac cycles were evaluated for each determination and heart rate (HR) was continuously monitored (8) (n = 9-15 animals/group; **Supplemental Table 2**).

**MI model and infarct size assessment**

Rat surgeries were conducted in a staggered schedule, with six procedures performed weekly following the order of animal acquisition. Group assignment was alternated across sessions to minimise procedural bias and ensure a uniform 14-day EPA-E treatment period. MI-induction and IS assessment were performed as previously described (9). Briefly, at week six, rats were anesthetized, intubated, and connected to a rodent ventilator. A thoracotomy was performed at the fourth intercostal space, and the LAD coronary artery was ligated for 45min. After this period, the animals were allowed to recover. 24h thereafter, the coronary artery was ligated at the same point as MI-induction, and 3mL of 1% Evans Blue (Evans Blue E2129-100G, Sigma-Aldrich) was perfused through the femoral vein to delineate the area at risk (AAR). Then, rats were euthanised by exsanguination through cardiac excision, and the heart was rapidly washed with PBS at 4°C and sliced into four 2-mm-thick sections. Slices were alternatively collected for molecular analyses and IS quantification [1% Triphenyl tetrazolium chloride staining] and histological studies (n = 11-14 animals/group; **Supplemental Table 2**).

Survival rates were assessed across two distinct phases: 1) the ischaemic phase, defined as the 45min LAD occlusion period; and 2) the acute post-MI phase, measured from LAD reperfusion to the time of sacrifice (24h post-MI) (n = 15-27 animals/group; **Supplemental Table 2**).

**Cardiac molecular fingerprint**

Ischaemic myocardium (delineated by Evans Blue negative staining) was collected and rapidly frozen in liquid nitrogen for mRNA (RNeasy Mini Kit, QIAGEN, Düsseldorf, Germany) and protein extraction (RIPA Lysis Buffer, Merck, Darmstadt, Germany). We assessed the entire ischaemic region, to determine the impact of EPA-E on the jeopardised myocardium (10,11).

*Transcript levels:* we determined cardiac gene expression levels of markers associated with 1) cellular apoptosis: *Becn1* (Rn00586976_m1); Cysteine-aspartic acid protease 3 (*Casp3*, Rn00563902_m1); 2) fibrosis: Collagen I (*Col1a1*, Rn0143848_m1) and Collagen III (*Col3a1* (Rn01437681_m1); and 3) inflammation: *Tlr4* (Rn00569848_m1) and mTOR (*Rptor* Rn01464431_m1). For this purpose*,* RNA concentration was determined with a NanoDrop ND-1000 spectrophotometer (NanoDrop Technologies; Thermo Fisher Scientific; Waltham, MA, USA) and purity was assessed by the A260/A280 ratio. cDNA was synthesised from 0.5 µg RNA using a reverse transcription kit (QIAGEN) and amplified by RT-PCR with the following specific probes (Applied Biosystems, ThermoFisher Scientific, Carlsbad, CA, USA). mRNA levels were normalised to the housekeeping gene HPRT 1 (Applied Biosystems). Each qPCR sample was analysed in duplicate, and the mean value was used for statistical analyses (n = 10-15 animals/group; **Supplemental Table 2**).

*Protein expression and activation:* we assessed myocardial markers of cellular apoptosis including cleaved caspase-3 (C-CASP3, MA1-91637, ThermoFisher Scientific) and total caspase-3 (Total-CASP3; #9662, Cell Signaling, Leiden, The Netherlands); cellular antioxidant capacity with glutathione peroxidase 1 (GPX1; ab22604, Abcam, Cambridge, United Kingdom); autophagy and lipid peroxidation by assessing phosphorylated protein kinase B (PKB) (p-AKT, #4060, Cell Signaling) and total AKT (#9272, Cell Signaling). We also determined mitochondrial homeostasis by measuring optic atrophy protein 1 (OPA1, #80471, Cell Signaling) and dynamin-related protein 1 (DRP1, #8570, Cell Signaling) protein levels. For the isolation of mitochondrial proteins, we followed the protocol described in (12). The intensity of the Western blot bands was assessed with Chemi-Doc (Bio-Rad, Life Science, Barcelona, Spain) and quantification was done with Image Lab Software (Bio-Rad, Life Science). Normalisation was performed against ponceau staining and the intensity of the bands was calculated by densitometry and expressed as arbitrary units (a.u.). Western blots were performed once per sample with equal protein loading; a common reference sample was included for inter-gel normalisation, and total protein levels were verified by Ponceau staining (n = 10-15 animals/group; **Supplemental Table 2**).

**Immunohistochemical analyses**

Paraffin-embedded slices from infarcted myocardial tissue of all animals were cut into 5μm thick serial sections (JUNG RM2055, Leica, Wetzlar, Germany) and processed for: 1) Masson’s trichrome staining (Bio-Optica, Milano, Italia). Preparations were scanned by Pannoramic MIDI 1.17 (3Dhistech) and images were acquired with the informatic program SlideViewer 2.7 (3Dhistech) and imported to ImageJ. The collagen volume fraction (CVF) was measured in five random fields per animal, as previously described (9); 2) lipid peroxidation was determined by measuring 4-Hydroxynonenal staining (4-HNE, 1:200, ab48506, Abcam) in ten random field; and, 3) innate immune cell recruitment by measuring total infiltrated neutrophils (anti-elastase; 1:200 ab68672-1001, Abcam), macrophages (anti-CD68; 1:200 ab31630, Abcam) and CD206^+^ macrophages (Anti-Mannose Receptor; 1:300, ab64693, Abcam). Positive cells were counted in ten random fields (n = 9-15 animals/group; **Supplemental Table 2**).

**Analyses of apoptosis execution by TUNEL fluorescence assay**

Apoptosis in the infarcted myocardial tissue was assessed using the TUNEL assay according to the manufacturer’s instructions (Chemicon Inc., Pittsburgh, PA, USA). Images were acquired using a Leica inverted fluorescence confocal microscope and quantified in four randomly selected fields per heart as previously described (9) (n = 11-14 animals/group; **Supplemental Table 2**).

**^1^H-NMR characterisation of** **the infarcted myocardial tissue and serum**

We carried out NMR spectroscopy of the infarcted myocardial tissue and serum samples to identify metabolic and lipid profile alterations associated with EPA-E administration. Aqueous and lipid extracts were obtained from infarcted myocardial tissue and serum using the Folch method with slight modifications (13). Briefly, 1440μL of dichloromethane:methanol (2:1, v/v) were added to 25mg of previously lyophilised tissue followed by three 5min sonication steps with one shaking step in between. Next, 400μL of ultrapure water was added, mixed and centrifuged at 25100g during 5min at 4ºC. Aqueous and lipid extracts were recovered and completely dried in Speedvac to achieve solvent evaporation and frozen at -80ºC until **^1^**H-NMR analysis.

Aqueous extracts were reconstituted in a solution of 45mM PBS containing 2.32mM of Trimethylsilylpropanoic acid (TSP) as a chemical shift reference and transferred into 5-mm NMR glass tubes. **^1^**H-NMR spectra were recorded at 300K operating at a proton frequency of 600.20MHz using an Avance III-600 Bruker spectrometer. One-dimensional 1H pulse experiments were carried out using 1D nuclear Overhauser effect spectroscopy with presaturation (NOESYPR1D) sequence to suppress the residual water peak at around 4.7ppm and a total of 64k data points were collected. The acquired spectra were phased, baseline-corrected and referenced before performing the automatic metabolite profiling of the spectra dataset. Low molecular weight metabolites (LMWMs) were identified and quantified using an adaptation of Dolphin (14). Several database engines (BBioref AMIX database (Bruker), Chenomx and HMDB (15), and literature (16) were used for 1D-resonances assignment and metabolite identification.

Lipid extracts were reconstituted in a solution of CDCl_3_:CD_3_OD:D_2_O (16:7:1, v/v/v) containing Tetramethylsilane (TMS) and transferred into 5-mm NMR glass tubes. Quantification of lipid signals in **^1^**H-NMR spectra was carried out using an adaptation of LipSpin (17). Resonance assignments were done based on literature values (16) (n = 8-11 animals/group; **Supplemental Table 2**). Remnant cholesterol (RM-C) serum levels were calculated as VLDL-C + IDL-C (mg/dL). The complete list of metabolites and lipid species analysed is presented in **Supplemental Table 3**.

**Lipoprotein analysis by NMR spectroscopy (advanced lipoprotein profile)**

We also obtained from serum the lipoprotein profile using the NMR test Liposcale ® (CE), as previously described (18). This protocol evaluates the lipid concentrations (i.e., TG and cholesterol), size and particle number of three different classes of lipoproteins (VLDL, LDL, and HDL), as well as the particle number of nine subclasses (large, medium, and small VLDL, LDL, and HDL). Briefly, particle concentration and diffusion coefficients were obtained from the measured amplitudes and attenuation of their spectroscopically distinct lipid methyl group NMR signals using the 2D diffusion-ordered 1H NMR spectroscopy (DSTE) pulse. The methyl signal was surface fitted with 9 lorentzian functions associated with each lipoprotein subclasses: large, medium and small of the main lipoprotein classes. The area of each lorentzian function was related to the lipid concentration of each lipoprotein subclass, and the size was calculated from their diffusion coefficient.

Each subclass particle concentration was calculated by dividing the lipid volume by the particle volume of a given class. Lipid volumes were determined by using common conversion factors to convert concentration units into volume units (19). The different lipoprotein subclasses correspond to the following diameter size ranges: large VLDL, 68.5 to 95.9nm; medium VLDL, 47 to 68.5nm; small VLDL, 32.5 to 47nm; large LDL, 24 to 32.5nm; medium LDL, 20.5 to 24nm; small LDL, 17.5 to 20.5nm; large HDL, 10.5 to 13.5nm; medium HDL, 8.5 to 10.5nm; and small HDL, 7.5 to 8.5nm. Finally, weighted average VLDL, LDL and HDL particle sizes were calculated from various subclass concentrations by summing the known diameter of each subclass multiplied by its relative percentage of subclass particle number.

**Targeted** **UHPLC-MS methodology for infarcted myocardial tissue**

200μL of lipidic extracts were dried under N_2_ and were reconstituted in 125μL of isopropanol.

Chromatographic analysis was performed on an Vanquish Horizon UHPLC (Thermo Scientific) coupled to a mass spectrometer Orbitrap ID-X Tribrid (Thermo Scientific). An Acquity UPLC BEH C18 (2.1 x 150mm, 1.7um) Waters. A gradient elution with (A) Acetonitrile:Water (60:40 v/v) containing 10mM ammonium acetate (B) Isopropanol:Acetonitrile (90:10 v/v) containing 10mM ammonium formate. The gradient program was as follows: 0-2min from 15% to 30% B, 2-2.5min 48% B, 2.5-8 82% B, 8-8.5 99% B, 8.5-9 99% B, 9-9.1 15% B, 9.1-12 15% B. The flow rate was set at 0.4ml/min. The injection volume was 2μL.

The ESI source was in negative ionisation mode. The optimal MS parameters were as follows: isolation mode in quadrupole; isolation window (m/z): 2; spray voltage: 3500V; sheath gas: 50; auxiliary gas: 10; ion transfer tube temperature: 300ºC; vaporiser temperature: 300ºC; orbitrap resolution: 60000; RF Lens (%): 60; AGC target: 2e5; Maximum injection time: Auto.

SIM mode using the precursors 303.2330 and 301.2173 was employed for the quantification of AA and EPA respectively. Relative quantification was performed by using Thermo Xcalibur QuanBrowser software. (n = 10-11 animals/group; **Supplemental Table 2**).

**Systemic cytokine levels**

We determined serological levels of Resolvin E1 (RvE-1; MBS2611781, MyBioSource, Inc, San Diego, USA) and IL-4 (E-EL-R0014, Elabscience, Texas, USA) with their respective enzyme-Linked Immunosorbent Assay kits (n = 8-9 animals/group; **Supplemental Table 2**).

**Biochemical and haematological follow-up**

Rats were deeply anesthetized (isoflurane, 26675-46-7, Zoetis, Parsippany, NJ, USA) at baseline, four, five and six weeks after dietary intervention and 24h post-MI for blood withdrawal and serum isolation (TP010350, Everest-Tecnovet, Barcelona, Spain). We assessed TG (GN 90125, RAL, Barcelona, Spain), total cholesterol (TC; GN 20125, RAL), and glucose (Accu-Chek InstantMOMG/DL, Roche Diabetes Care, Barcelona, Spain) levels (n = 12-15 animals/group; **Supplemental Table 2**). Serum non-esterified fatty acids (NEFA) were quantified using an enzymatic colourimetric assay (NEFA-HR(2), Wako Chemicals GmbH, Neuss, Germany) according to the manufacturer's instructions (n = 9-11 animals/group; **Supplemental Table 2**).

**REFERENCES**

1.	du Sert NP, Ahluwalia A, Alam S, Avey MT, Baker M, Browne WJ, et al. Reporting animal research: Explanation and elaboration for the arrive guidelines 2.0. Vol. 18, PLoS Biology. 2020. 1–65 p.

2.	Bøtker HE, Hausenloy D, Andreadou I, Antonucci S, Boengler K, Davidson SM, et al. Practical guidelines for rigor and reproducibility in preclinical and clinical studies on cardioprotection [Internet]. Vol. 113, Basic Research in Cardiology. Springer Berlin Heidelberg; 2018. 1–73 p. Available from: https://doi.org/10.1007/s00395-018-0696-8

3.	Lindsey ML, Bolli R, Canty JM, Du XJ, Frangogiannis NG, Frantz S, et al. Guidelines for experimental models of myocardial ischemia and infarction. Am J Physiol - Hear Circ Physiol. 2018;314(4):H812–38.

4.	Mach F, Baigent C, Catapano AL, Koskinas KC, Casula M, Badimon L, et al. 2019 ESC/EAS Guidelines for the management of dyslipidaemias: lipid modification to reduce cardiovascular risk: The Task Force for the management of dyslipidaemias of the European Society of Cardiology (ESC) and European Atherosclerosis Society (EAS). Eur Heart J. 2020;41(1):111–88.

5.	Laufs U, Parhofer KG, Ginsberg HN, Hegele RA. Clinical review on triglycerides. Eur Heart J. 2020 Jan;41(1):99-109c.

6.	Klimes I, Sebokova E, Zicha J, Vrána A, Kunes J, Seböková E, et al. Hereditary hypertriglyceridemic rat: a new animal model of metabolic alterations in hypertension. Blood Press. 1995 May;4(3):137–42.

7.	Bhatt DL, Steg PG, Miller M, Brinton EA, Jacobson TA, Ketchum SB, et al. Cardiovascular Risk Reduction with Icosapent Ethyl for Hypertriglyceridemia. N Engl J Med. 2019;380(1):11–22.

8.	Zacchigna S, Paldino A, Falcão-Pires I, Daskalopoulos EP, Dal Ferro M, Vodret S, et al. Towards standardization of echocardiography for the evaluation of left ventricular function in adult rodents: a position paper of the ESC Working Group on Myocardial Function. Cardiovasc Res [Internet]. 2021 Jan 1;117(1):43–59. Available from: https://academic.oup.com/cardiovascres/article/117/1/43/5828935

9.	Alcover S, López S, Ramos L, Muñoz-García N, Gallinat A, Suades R, et al. Cardioprotection during myocardial infarction in diabetic cardiomyopathy. Diabetes [Internet]. 2025 Mar 13;74(6):db240510. Available from: https://doi.org/10.2337/db24-0510

10.	Chen B, Frangogiannis NG. Chemokines in Myocardial Infarction. J Cardiovasc Transl Res [Internet]. 2021;14(1):35–52. Available from: https://doi.org/10.1007/s12265-020-10006-7

11.	Daseke MJ 2nd, Tenkorang MAA, Chalise U, Konfrst SR, Lindsey ML. Cardiac fibroblast activation during myocardial infarction wound healing: Fibroblast polarization after MI. J Int Soc Matrix Biol. 2021 Sep;91–92(1):109–116.

12.	Kobara M, Shiraishi T, Noda K, Toba H, Nakata T. Eicosapentaenoic Acid Preserves Mitochondrial Quality and Attenuates Cardiac Remodeling After Myocardial Infarction in Rats. J Cardiovasc Transl Res [Internet]. 2023;16(4):816–27. Available from: https://doi.org/10.1007/s12265-023-10363-z

13.	FOLCH J, LEES M, SLOANE STANLEY GH. A simple method for the isolation and purification of total lipides from animal tissues. J Biol Chem. 1957 May;226(1):497–509.

14.	Gómez J, Brezmes J, Mallol R, Rodríguez MA, Vinaixa M, Salek RM, et al. Dolphin: a tool for automatic targeted metabolite profiling using 1D and 2D (1)H-NMR data. Anal Bioanal Chem. 2014 Dec;406(30):7967–76.

15.	Wishart DS, Feunang YD, Marcu A, Guo AC, Liang K, Vázquez-Fresno R, et al. HMDB 4.0: the human metabolome database for 2018. Nucleic Acids Res. 2018 Jan;46(D1):D608–17.

16.	Vinaixa M, Rodríguez MA, Rull A, Beltrán R, Bladé C, Brezmes J, et al. Metabolomic assessment of the effect of dietary cholesterol in the progressive development of fatty liver disease. J Proteome Res. 2010 May;9(5):2527–38.

17.	Barrilero R, Gil M, Amigó N, Dias CB, Wood LG, Garg ML, et al. LipSpin: A New Bioinformatics Tool for Quantitative (1)H NMR Lipid Profiling. Anal Chem. 2018 Feb;90(3):2031–40.

18.	Mallol R, Amigó N, Rodríguez MA, Heras M, Vinaixa M, Plana N, et al. Liposcale: a novel advanced lipoprotein test based on 2D diffusion-ordered 1H NMR spectroscopy. J Lipid Res. 2015 Mar;56(3):737–46.

19.	Jeyarajah EJ, Cromwell WC, Otvos JD. Lipoprotein particle analysis by nuclear magnetic resonance spectroscopy. Clin Lab Med. 2006 Dec;26(4):847–70.
